# Variation in responses to temperature in admixed *Populus* genotypes predicts geographic shifts in regions where hybrids are favored

**DOI:** 10.1111/nph.70787

**Published:** 2025-11-30

**Authors:** Alayna Mead, Joie R. Beasley‐Bennett, Andrew Bleich, Dylan Fischer, Shelby Flint, Julie Golightly, Lee Kalcsits, Sara K. Klopf, Mason W. Kulbaba, Jesse R. Lasky, Jared M. LeBoldus, David B. Lowry, Nora Mitchell, Emily Moran, Jason P. Sexton, Kelsey L. Søndreli, Baxter Worthing, Michelle Zavala‐Paez, Matthew C. Fitzpatrick, Jason Holliday, Stephen Keller, Jill A. Hamilton

**Affiliations:** ^1^ Pennsylvania State University University Park PA 16802 USA; ^2^ Oregon State University Corvallis OR 97331 USA; ^3^ Michigan State University East Lansing MI 48824 USA; ^4^ The Evergreen State College Olympia WA 98505 USA; ^5^ Southwest Minnesota State University Marshall MN 56258 USA; ^6^ Salisbury University Salisbury MD 21801 USA; ^7^ Washington State University Wenatchee WA 98801 USA; ^8^ Virginia Tech Blacksburg VA 24061 USA; ^9^ St. Mary's University Calgary AB T2X 1Z4 Canada; ^10^ Pennsylvania State University University Park PA 16802 USA; ^11^ University of Wisconsin – Eau Claire Eau Claire WI 54701 USA; ^12^ University of California, Merced Merced CA 95343 USA; ^13^ University of Vermont Burlington VT 05405 USA; ^14^ University of Maryland Center for Environmental Science Frostburg MD 21532 USA

**Keywords:** climate change, climate response function, hybrid zone, plasticity, *Populus*, provenance trial

## Abstract

- Plastic responses of plants to their environment vary as a result of genetic differentiation within and among species. To accurately predict rangewide responses to climate change, it is necessary to characterize genotype‐specific reaction norms across the continuum of historic and future climate conditions comprising a species' range.
- The North American hybrid zone of *Populus trichocarpa* and *Populus balsamifera* represents a natural system that has been shaped by climate, geography, and introgression. We leverage a dataset containing 44 clonal genotypes from this natural hybrid zone, planted across 17 replicated common garden experiments spanning a broad climatic range. Growth and mortality were measured over 2 yr, enabling us to model reaction norms for each genotype across these tested environments.
- Species ancestry and intraspecific genomic variation significantly influenced growth across environments, with genotypic variation in reaction norms reflecting a trade‐off between cold tolerance and growth. Using modeled reaction norms for each genotype, we predicted that genotypes with more *P. trichocarpa* ancestry may gain an advantage under warmer climates.
- Spatial shifts of the hybrid zone could facilitate the spread of beneficial alleles into novel climates. These results highlight that genotypic variation in responses to temperature will have landscape‐level effects.

## Resumen

- Las respuestas plásticas de las plantas a su entorno varían como resultado de la diferenciación genética entre y dentro de las especies. Para predecir con exactitud las respuestas de una especie al cambio climático a lo largo de su rango de distribución, es necesario caracterizar las normas de reacción específicas de cada genotipo a lo largo del continuo de condiciones climáticas históricas y futuras que conforman su rango de distribución.
- La zona híbrida norteamericana de *Populus trichocarpa* y *Populus balsamifera* representa un sistema natural que ha sido estructurado por el clima, la geografía y la introgresión. Utilizamos un conjunto de datos con 44 genotipos clonales de esta zona híbrida natural, establecidos en 17 jardines comunes replicados que cubren un amplio gradiente climático. El crecimiento y la mortalidad se midieron durante dos años, lo que nos permitió modelar las normas de reacción de cada genotipo en los distintos ambientes evaluados.
- La ancestría de especie y la variación genómica intraespecífica influyeron significativamente en el crecimiento a través de los distintos ambientes, con la variación genotípica en las normas de reacción reflejando una compensación entre la tolerancia al frío y el crecimiento. A partir de las normas de reacción modeladas para cada genotipo, se predijo que los genotipos con mayor ascendencia de *P. trichocarpa* podrían tener una ventaja en climas más calientes.
- Los cambios espaciales de la zona híbrida podrían facilitar la propagación de alelos beneficiosos hacia climas nuevos. Estos resultados destacan que la variación genotípica en las respuestas a la temperatura tendrá efectos a escala de paisaje.

## Introduction

Predicting the phenotypic responses of populations to changing climates and how they vary across species ranges is essential for conserving and restoring native ecosystems. Predicted changes in fitness between current and future climates can be used to identify populations most at risk, or to identify populations resilient to change which could be valuable seed sources for assisted gene flow and restoration (Aitken & Whitlock, 2013; Aitken & Bemmels, 2016). Phenotypes expressed in the field arise from the genotypic variation underlying phenotypic traits (*G*), the plastic response to the environment (*E*), and genotypic variation in response to the environment (*G* × *E*) (Des Marais *et al*., 2013). Each of these factors varies across complex and changing landscapes. By characterizing reaction norms, the set of phenotypes expressed across a range of environments, it is possible to predict organisms' responses to the variable environments they inhabit and understand the selective forces that may be shaping plasticity (Via *et al*., 1995; Arnold *et al*., 2019).

Reaction norms can vary within species, resulting in part from selection imposed by spatially varying climates (Des Marais *et al*., 2013; Ikeda *et al*., 2017; Rehfeldt *et al*., 2018; Patsiou *et al*., 2020). Predicting genotype‐specific responses will be particularly important for species with large ranges, which may exhibit variation in reaction norms due to the combined influence of geography, population demography, and climate (Cooper *et al*., 2019, 2022; Van Nuland *et al*., 2020). This is particularly true where glacial refugia have shaped species' demographic history and connectivity (Woolbright *et al*., 2014; Love *et al*., 2023; Bolte *et al*., 2024). However, because of the difficulty of phenotyping multiple genotypes across many environments, many methods for modeling a species' response to climate change ignore genotypic variation, and instead consider only the climate envelope of the extant species range (Capblancq *et al*., 2020). Furthermore, regions within a species' range will vary in the rate, magnitude, and nature of climate change (e.g. increases or decreases in precipitation), making it important to understand localized climate responses. To accurately predict local phenotypic responses to changing climates, it is necessary to test whether reaction norms vary across genotypes, and if so, to characterize genotype‐specific reaction norms across the continuum of historic and future climate conditions comprising a species' range (Arnold *et al*., 2019; VanWallendael *et al*., 2022).

Common garden experiments and provenance trials are invaluable tools for quantifying intraspecific variation in phenotypic plasticity and for predicting phenotypic responses to current and future environments (O'Neill *et al*., 2008; Wang *et al*., 2010; Leites *et al*., 2012; Fischer *et al*., 2014; Grady *et al*., 2015; Browne *et al*., 2019; Leites & Benito Garzón, 2023; Ye *et al*., 2023; Hord *et al*., 2025). Variation in reaction norms across genetic and climatic gradients can be quantified using repeated plantings of genotypes in multiple environments. Genotype‐specific reaction norms modeled across continuous environments are used to identify genotypes or loci associated with optimized fitness or yield across varying environmental conditions; however, such studies have been limited to a handful of species (Gray *et al*., 2011; Arnold *et al*., 2019; Lowry *et al*., 2019; Patsiou *et al*., 2020; VanWallendael *et al*., 2022; Li *et al*., 2025 ). Because plasticity results from adaptation to climate as well as demographic history, it may be possible to predict the reaction norms of unmeasured genotypes if they vary as a function of climate of origin (O'Neill *et al*., 2008; Wang *et al*., 2010; Rehfeldt *et al*., 2018) or genetic variation. When genomic data are available in addition to phenotypic and climate data collected in common garden experiments, predictions of phenotypic responses can be improved (Mahony *et al*., 2020; Archambeau *et al*., 2022; Putra *et al*., 2023; Li *et al*., 2025). Therefore, incorporating genetic information into characterization of reaction norms will enable more accurate predictions of rangewide climate responses.

Natural hybrid zones are ideal systems for disentangling the effects of adaptation and demographic history on phenotypes, including reaction norms, because multiple generations of backcrossing can result in novel recombinant genotypes associated with high phenotypic variability (Janes & Hamilton, 2017). Hybrid zones can increase the genetic variation available for adaptation to rapidly changing climates and allow movement of adaptive loci from one species into another via introgression (Janes & Hamilton, 2017; Suarez‐Gonzalez *et al*., 2018b; Kremer & Hipp, 2020; Buck *et al*., 2023; Hord *et al*., 2025). Comparing responses to the environment across admixed genotypes reveals how existing genetic variation within hybrid zones underlies fitness differences across environments. Hybrid zones can also be used to monitor responses to climate change (Taylor *et al*., 2015), with documented geographic shifts in some zones in response to changing environments (Billerman *et al*., 2016; Wielstra, 2019; Alexander *et al*., 2022). If reaction norms depart from the intraspecific pattern across a hybrid zone, climate change may alter regions where particular species, genotypes, or even genes are favored (Hord *et al*., 2025).

**Fig. 1:**
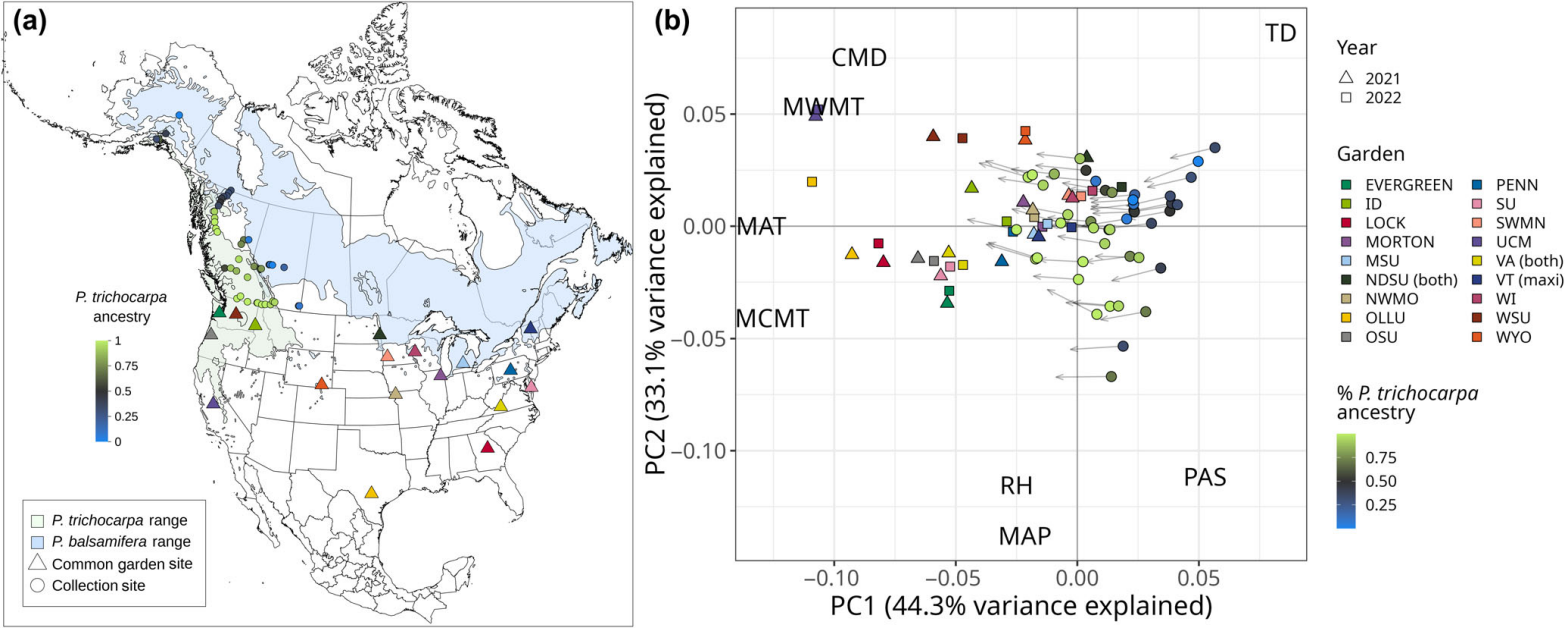
Studied genotypes were collected from the hybrid zone between *Populus balsamifera* and *Populus trichocarpa*, then planted into common gardens throughout the United States, including environments warmer than their home climate. (a) Map of sampled genotype localities (circles) and common garden sites (triangles) in relation to the ranges of both species (Little, 1971). The color of sampled genotypes indicates the proportion of species ancestry calculated from Admixture at *K* = 2, with blue indicating a higher proportion of *P. balsamifera* ancestry and green indicating higher *P. trichocarpa* ancestry. Garden sites indicate mini gardens, unless noted in the legend as having a maxi garden or both mini and maxi gardens. (b) Principal component analysis of past and future climates across collection and garden sites. Gardens were generally warmer than home sites (lower principal component 1 (PC1) loadings), and their position in multivariate climate space in PC1 and PC2 overlapped with the expected future climates for some genotypes. Circles represent the historic climate (1961–1990) of each genotype's provenance, colored by species ancestry, and arrowheads represent the predicted climate for 2041–2070 under an ensemble of 13 general circulation models. Triangles and squares represent the climate at common garden sites for 2021 and 2022. Text indicates the loadings of climate variables on each axis, with abbreviations as follows: CMD, climatic moisture deficit; MAP, mean annual precipitation; MAT, mean annual temperature; MCMT, mean coldest month temperature; MWMT, mean warmest month temperature; PAS, precipitation as snow; RH, relative humidity; TD, temperature difference, or continentality. Loadings of climate variables have been downscaled to allow visibility of sites.

North American *Populus* is a model system for forest trees due to their small genomes, ease of clonal propagation, and development as a biofuel feedstock (Jansson & Douglas, 2007; Sannigrahi *et al*., 2010; Porth & El‐Kassaby, 2015). Yet, like many nonmodel trees, its genetic variation is shaped by interactions between climate, geography, and interspecific introgression. *Populus* species occupy heterogeneous landscapes, often forming multi‐species hybrid zones that are a source of novel recombinant genetic variation (Suarez‐Gonzalez *et al*., 2016, 2018a; Chhatre *et al*., 2018; Bolte *et al*., 2024). These factors make *Populus* an ideal system for quantifying intraspecific variation in climate responses and predicting landscape‐scale changes in fitness. Here, we focus on the North American hybrid zone between *Populus trichocarpa*, a western species spanning latitudes from Alaska to California, and *Populus balsamifera*, which occurs transcontinentally throughout the boreal regions of the contiguous United States, Canada, and Alaska (Fig. 1a). Populations within the hybrid zone exhibit genetic and phenotypic differentiation, likely resulting from varying demographic history as well as adaptation to wide‐ranging biotic and abiotic environments (Keller *et al*., 2010, 2011, 2012; Slavov *et al*., 2012; Evans *et al*., 2014; Geraldes *et al*., 2014; McKown *et al*., 2014a,b,c; Zhou *et al*., 2014; Fitzpatrick & Keller, 2015; Holliday *et al*., 2016; Chhetri *et al*., 2019; Zhang *et al*., 2019; Fitzpatrick *et al*., 2021). Gene flow rates across the hybrid zone vary in part due to geographic barriers, resulting in spatial differences in population differentiation (Bolte *et al*., 2024).

We leveraged the *P. trichocarpa* × *P. balsamifera* hybrid zone to determine how genetic and environmental parameters interact to influence genotype‐specific reaction norms, ultimately using our predictions to model changes to hybrid zone composition in the context of global change. We used a series of replicated provenance trials of clonal genotypes from the hybrid zone, which we planted in 17 gardens across the United States that span a wide range of environments, including many warmer than the climate of origin. We tested three hypotheses: Hypothesis 1: *Populus* hybrids display heritable differences in adaptation to climate, which manifest as genotype‐specific responses to climatic variation. To test this, we measured fitness‐related traits in each garden to determine how genetic background and the environment interact to determine fitness in this system, quantifying the effect of genetic structure (genotype effect, *G*), climate (environmental effect, *E*), and how the response to climate varies by genotype (*G* × *E*). A significant genotype × environment interaction could suggest that the response to climate is mediated by local adaptation to the climate of origin. Hypothesis 2: Variation in reaction norms is predictable based on a continuous gradient of genomic ancestry, enabling us to predict where the hybrid zone is favored to move under future climates. To test this, we used whole‐genome sequence data to characterize multivariate genetic structure, including both species ancestry and intraspecific variation, and predicted its effect on phenotypic responses among genotypes. We developed a model to predict growth and mortality for a given garden environment using information about each genotype's climate of origin and genetic data, which was validated using a combination of 500 novel genotypes and novel environments. Finally, we used genotype‐specific reaction norms to predict phenotypic responses to climate change across the landscape of the hybrid zone. Hypothesis 3: Inclusion of genetic information in phenotypic response models improves their predictions due to inherent genetic structure contributing to variation in reaction norms. We tested whether the climate of origin can serve as a proxy for adaptive genetic structure, circumventing the need for genetic data.

## Materials and Methods

### Field collections and propagation

In 2020, we established 17 common gardens containing 48 clonally replicated poplar genotypes, including *Populus trichocarpa* Torr. & A. Gray, *Populus balsamifera* L., and admixed individuals. Clonal replicates were propagated from dormant vegetative cuttings taken from 48 different mature trees in the wild between October 2019 and March 2020. These genotypes originated from natural populations spanning five transects that traverse the natural hybrid zone of *P. trichocarpa* and *P. balsamifera*, including latitudes from Alaska to southern British Columbia and Alberta (Fig. 1). Dormant vegetative cuttings were transported to Virginia Tech (Critz, VA, USA) for propagation, as described in Bolte *et al*. (2024). Briefly, cuttings were exposed to a 30‐s ZeroTol 2.0 fungal dip treatment, dipped in Garden Safe Take Root hormone (0.1% indole‐3‐butyric acid), rooted into a standard rooting mix, and placed on a mist bench for 38 d following which clonal genotypes were shipped and planted into common garden sites.

### Common garden design

Seventeen common gardens were established at colleges, universities, and arboreta across the United States in fall 2020 (Fig. 1a; Supporting Information Table S1). We visualized variation in climate space among garden environments and past and future home environments using a principal component analysis (PCA) of environmental variables (Fig. 1b). Garden sites span a range of environments and include the southern range of both *P. trichocarpa* and *P. balsamifera*, as well as warmer regions to the south of their native ranges, enabling us to predict responses to novel climates (Fig. 1b). Each garden included two blocks, with one individual per genotype planted across each block in a randomized complete block design. In total, the design included 48 genotypes × 17 gardens × 2 blocks × 1 genotype per block. Each garden had a minimum of 94 trees, for a total of 1656 individuals included across all gardens. Phenotypic data were collected during the growing season for 3 yr: 2021–2023. All 17 gardens were evaluated in the first year, but high mortality and changing local phenotyping capacity at some sites decreased the total number of gardens assessed to 15 and 12 sites in 2022 and 2023, respectively (Table S1).

As part of the same experiment, three additional common gardens were established in March 2020. These gardens included 544 genotypes, each with three clonally replicated individuals planted in three blocks (one replicate per block), located at North Dakota State University (NDSU), Virginia Tech University (VA), and the University of Vermont (VT). We designated the 17 smaller gardens as ‘mini’ gardens, and the three larger gardens as ‘maxi’ gardens. All 48 genotypes included in the mini gardens were also planted in the maxi gardens. Here, we focus on phenotypic variation across the mini garden environments and use the maxi gardens for model evaluation (see ‘Statistical model’ in the Materials and Methods section).

### Phenotypic data

Height was measured each year (2021–2023) before bud flush and after budset. Annual growth increment was calculated per year based on the height accumulated during the growing season (pre‐bud flush height subtracted from post‐budset height). Some gardens experienced herbivory, disease, and accidental mechanical damage leading to negative growth increments for some individuals. As these negative growth increments likely represent the consequences of measurement error or herbivory, they were removed before analyses (137 individuals in 2021 and 97 in 2022). Our goal was to isolate the effects of climate on growth. While the impacts of herbivory will also likely shift with climate change, they may vary outside of the native range, and herbivory also likely differed across gardens for nonclimatic reasons (e.g. fencing and human presence), so we excluded its effects here. Given the frequency of herbivory, it is likely that some growth increment values are underestimates of potential yearly growth.

### Climate data

Climate data were extracted from ClimateNA rasters (Wang *et al*., 2016; AdaptWest Project, 2022) using terra 1.8‐21 (Hijmans, 2025) in R v.4.4.2 (R Core Team, 2024) for genotype provenances (locations of origin) and common garden environments. For provenances, we extracted historical climate data for the 30‐yr period from 1961 to 1990. We focused on average historical climate data because variation in climate response is partially the result of selection associated with the climate of origin during establishment and over the lifespan of the collected adult trees, which average > 30 yr old. For garden climates, we extracted the climate averages associated with each year of data collection, starting when the seedlings were planted in 2020. We also fit the model (to be described later) with 2021 data using 22 climate variables as the home and garden climates, dropping the block effect to allow all models to converge, and compared their Akaike information criterion (AIC) scores. We selected mean coldest month temperature (MCMT) for use in statistical modeling because it explained variation in performance across gardens and had the lowest AIC score (Table S2). Moreover, the range of MCMT values at the common garden sites encompassed climates for most natural populations (see the Results section). This enabled us to predict growth and mortality responses to MCMT within much of the native range of the two poplar species. We did not use precipitation variables in statistical modeling because most gardens were irrigated during the first year, and some drier gardens (OLLU, SWMN, and UCM) continued irrigation in later years to ensure survival, limiting the selective effect of precipitation variation across gardens.

### Genomic data

Whole‐genome sequence data associated with each genotype and previously described in Bolte *et al*. (2024) was used in phenotypic prediction across common gardens. For each genotype, *c*. 100 mg of young leaf tissue was used for genomic DNA extraction with the Qiagen Plant DNeasy Kit. Genomic libraries were sequenced using an Illumina NovaSeq 6000, with 64 samples per lane, using paired‐end 150‐bp reads. Reads were aligned to the *P. trichocarpa* reference genome (v.4.0) and variant calling was performed using Gatk Haplotype Caller. Within Gatk, variants were filtered for quality‐by‐depth (QD < 2), mapping quality (MQ < 40), elevated strand bias (FS > 40, SOR > 3), and differential map quality and positional bias between reference and alternate alleles (MQRankSum < −12.5, ReadPosRankSum < −8). Using Bcftools, variants were subset to biallelic single nucleotide polymorphisms (SNPs) (‐m2 ‐M2 snps) and variants with a minor allele count of 1 (‐‐include ‘MAC > 1’). Vcftools was used to remove all SNPs with missing data across individuals (max‐missing 1.0). Finally, variants were pruned for linkage disequilibrium (LD) in Plink using a 10 000‐bp window size, shifted by 1000 bp, removing variants with a pairwise *R* ^2^ value > 0.1. Filtering scripts are available at https://github.com/alaynamead/poplar_hybrid_vcf_filtering. After filtering and LD‐pruning, a total of 334 657 variable sites were used to characterize genetic variation and admixture.

Bolte *et al*. (2024) identified three distinct lineages in this hybrid zone: *P. balsamifera*, coastal *P. trichocarpa*, and a separate interior *P. trichocarpa* lineage originating from an ancient admixture event between the two species. We incorporated this genetic variation into our predictions using the principal component (PC) scores of each genotype from a PCA of SNP data created with vegan v.2.6‐8 in R (Oksanen *et al*., 2024), defining genetic structure as variation in genomic PC space. Principal component 1 (PC1) separates *P. balsamifera* and the two *P. trichocarpa* lineages, PC2 separates all three lineages, and PC3 separates the northern (Alaska and Cassiar) and southern (Chilcotin, Jasper, and Crowsnest) transects (Fig. S1). To visualize how responses varied by species ancestry, we used ancestry proportions based on *K* = 2 from the ancestry estimation software Admixture (Alexander *et al*., 2009). Across 48 genotypes, four genotypes were previously identified as genetic outliers, possibly as a result of mixed ancestry with additional *Populus* species (Bolte *et al*., 2024; Fig. S2). These individuals were removed from the analyses, leaving a total of 44 genotypes for statistical modeling.

### Statistical model

We fit a generalized linear mixed model to evaluate the effects of garden climate, home climate, genetic structure and their interactions (*G* × *E*) on growth across the common gardens and to predict responses to future climates. We defined environment as the MCMT of each garden during the year of measurement, and we defined home climate as the historical average of MCMT (1961–1980) at the provenance origin for each genotype. Genotypes may vary in their responses to climate as a result of local adaptation to their climate of origin and as a result of neutral genetic variation. Given this, we accounted for these sources of variation using different model variables. Previous studies have tested how climate of origin determines genotypic variation in responses to the planting environment, assuming that responses are largely explained by local adaptation and modeling *G* × *E* using climate of origin as a proxy for genetic variation (O'Neill *et al*., 2008; Wang *et al*., 2010). However, this approach does not account for variation in responses that result from other processes, such as neutral evolution resulting from historic demographic processes, selection for recent but not long‐term climate patterns, or novel recombinants originating from hybridization. We include both genetic structure and climate of origin in the model to account for these multiple interacting factors that may influence the response to the environment.

We included data from the first 2 yr of measurement (2021 and 2022) across 17 and 14 gardens, respectively, analyzing 2768 measurements of 1610 individual trees. We excluded 2023 data because gardens at the upper and lower temperature extremes were lost due to high mortality (Fig. S2), and climatic extremes are important to provide ‘anchor points’ to produce biologically realistic response curves (Wang *et al*., 2006). However, we did compare the model fit for each year individually and observed similar effect directions across all 3 yr.

We included survival and yearly growth increment as two measures of performance within the same model by fitting a zero‐inflated Gaussian model using glmmtmb v.1.1.11 (Brooks *et al*., 2017) in R v.4.5.1 (R Core Team, 2024). We set the growth of all trees marked dead at the end of each growing season as 0, resulting in a large number of zero values (381 trees in 2021 and 343 trees in 2022, 27% of the total). This model assumes that values of zero could have two origins, which were modeled as two separate components within one model. The first ‘conditional’ component modeled ‘sampling’ zeros as part of the continuous distribution of growth increment (i.e. some individuals survived but had growth at or near zero). The second ‘zero‐inflated’ component modeled ‘structural’ zeros attributed to mortality. These are modeled by the zero‐inflated component, taking binary mortality values as input to predict the probability of mortality using a logit link function (Hu *et al*., 2011; Brooks *et al*., 2017). This method allowed us to evaluate how two fitness‐associated traits, growth and mortality, separately respond to MCMT. Together, their combined effect can be considered a proxy for overall fitness, incorporating both the probability of mortality and the predicted growth for surviving individuals. We report phenotypic predictions for three model components: the zero‐inflated component representing mortality, the conditional component representing growth, and the overall model combining the two.

The full model included the fixed effects of garden MCMT, home MCMT, their square terms (to account for the observed quadratic response associated with a fitness optimum; Figs S2, S3), and all interactions between garden and home MCMT and their square terms (indicating that the response to garden temperature varies based on the temperature of origin). We also included population genetic structure as a fixed effect using the values for genetic PC1, PC2, and PC3 (Fig. S1). MCMT was negatively correlated with genetic PC1 (*R* = −0.69), consistent with the climate niches associated with the two species, but was only weakly correlated with PC2 (*R* = −0.22) and PC3 (−0.40), so including multiple PCs enables us to identify variation in responses resulting from neutral genetic structure or adaptation to other climate variables. We tested for *G* × *E* by including interaction effects between garden climate and the genetic PCs. We included the random intercepts of individual, genotype, year, and block nested within garden. The same formula was used for the zero‐inflated portion of the model to test the effect of each factor on mortality. To improve model convergence, we scaled the numeric variables using the R function scale without centering and back‐transformed scaled values to actual values for visualization. Growth increment was log‐transformed to account for a distribution skewed toward lower values. Using each individual as a separate observation, we fit the following model using the glmmtmb function, accounting for home MCMT, garden MCMT and their interactions and as well as genetic PCs 1–3 and their interaction terms with garden MCMT. This modeled the *j*th individual at the *i*th garden, where *μ* represents the intercept, *H*_*j*_ is the home environment and *G*_*i*_ is the garden environment *i*; *A*_*j*_, *B*_*j*_, and *C*_*j*_ are, respectively, genetic PCs 1, 2, and 3 for individual *j*; block, genotype, year, and individual are random intercepts, and *ɛ* represents the error term.

**Model 1:**

$$\begin{array}{c} Y_{\text{ijklm}}=\mu +G_{i}^{2}+G_{i}+H_{j}^{2}+H_{j}+G_{i}^{2}H_{j}^{2}+G_{i}^{2}H_{j}+G_{i}H_{j}^{2}\;+\;G_{i}H_{j}+A_{j}+B_{j}+C_{j}+A_{j}G_{i}+A_{j}G_{i}^{2}+B_{j}G_{i}+B_{j}\;+\;G_{i}^{2}+C_{j}G_{i}+C_{j}G_{i}^{2}+{\text{block}}_{i\left(k\right)}+{\text{genotype}}_{l}\;+\;{\text{year}}_{m}+{\text{indiv}}_{i}+\epsilon_{\text{ijklm}} \end{array}$$

The significance of each term was evaluated using the tab_model function from the R package sjplot v.2.8.17 (Lüdecke, 2024), which uses a type II Wald chi‐squared test. Estimates for each factor were standardized by dividing by its SD using tab_model and plot_model functions from sjplot to facilitate comparisons between the relative effect sizes of linear and quadratic terms and their interactions.

### Model evaluation

To evaluate whether Model 1 could accurately predict poplar phenotypic response to environmental conditions, we compared its predictions of growth and mortality to actual measures in two ways. For both methods, we predicted the growth increment and mortality for each tree, using the garden MCMT and each genotype's home MCMT values and genomic PC values as model parameters. First, we removed one of the garden environments from the dataset, trained the model on the remaining 16 gardens, and used the fitted model to predict growth increment in the garden that was excluded, repeating this process for each garden (hereafter referred to as leave‐one‐out cross‐validation). Second, we trained the model using all 17 gardens and then predicted growth increments of 544 genotypes in the three maxi gardens. Two maxi garden sites also have a mini garden site that was included in the training data (Virginia and North Dakota), and one is a novel environment without a mini garden (University of Vermont). Five hundred of these genotypes were novel genotypes not evaluated within the mini gardens, and we used their genomic PC values and home climate to predict their responses to garden climate. We predicted growth increments for individuals based on the full model using the predict function from the glmmtmb package and calculated the Pearson correlation between the actual values and the predicted values. We also tested whether the estimated probability of mortality corresponded to measured mortality rates using a generalized linear model with a binomial link function. Full details of the methods used to predict phenotypes are included in Methods S1.

We used the same leave‐one‐out cross‐validation method to compare the performance of three different models with different combinations of genetic and climatic information. If most of the variation among genotypes in response to garden MCMT is explained by local adaptation, provenance MCMT could serve as a proxy for genetic structure, enabling genotype‐specific predictions of responses to temperature without the need for genetic data. To determine whether genetic PCs or home temperature increased the predictive power of the model, we tested simplified versions of the full model described above: Model 2, a genetics‐only model with home temperature excluded to test the predictive power of the genetic PCs, and Model 3, a temperature‐only model with genetic information excluded to test the predictive power of home MCMT (Table 1). We trained the three models on datasets with each garden excluded and predicted responses in the excluded garden for 2021 and 2022, evaluating models using the Pearson correlation between actual and predicted growth increments as described previously. We tested whether the models differed in predictive ability using an ANOVA, with the 17 cross‐validation tests used as replicates.

**Table 1:** Factors included in three separate models predicting growth and mortality in *Populus trichocarpa*, *Populus balsamifera*, and admixed genotypes.

	*E*: garden MCMT (quadratic)	*G*: genetic PCs 1–3	Genotype × garden MCMT (*G* × *E*)	Home MCMT × garden MCMT (quadratic) (*G* × *E* proxy)
Model 1: Full	×	×	×	×
Model 2: Genetics only	×	×	×	
Model 3: Climate only	×			×

Models were compared to test whether home climate can be a proxy for genetic structure in modeling the response to the environment. Model 1 is the full model and includes the effects of environment (*E*), genetics (*G*), and their interaction (*G* × *E*), Model 2 drops the effect of home climate, and Model 3 drops the effect of genetics. MCMT, mean coldest month temperature.

### Predicting the response to temperature

We used the parameters estimated from the full model (Model 1) to predict each genotype's norm of reaction, that is the response to a continuous temperature gradient, allowing us to characterize responses to temperature across the hybrid zone. We predicted the growth increment and mortality probability of each genotype across a range of 100 equally spaced MCMT values from −23.9°C to 9.8°C, encompassing both historic 30‐yr home climates (−23.9°C to −3.8°C) and yearly garden climates (−16.5°C to 9.8°C). Phenotypic predictions should be more reliable for the warmer climatic range included in the common gardens where phenotypic data were collected than for colder regions, enabling us to predict responses to warming climates. We predicted the phenotypic response of each genotype across this climatic range of MCMT, accounting for genotype‐specific responses with the genetic PCs and the home MCMT of that genotype, using the parameters estimated from Model 1. The glmmtmb predict function was used, ignoring the random effects of genotype, block, and garden and predicting the overall response to temperature rather than the genotype‐ or garden‐specific response (option re.form = NA). Phenotypic predictions were made for the two model components and for their combined effect: the conditional component reflecting growth increment, the zero‐inflated component predicting the probability of mortality, and the combined model incorporating both. We also calculated the climate transfer distance as the difference between garden and home MCMT for each genotype, and plotted reaction norms for each genotype against this value to visualize where maximum growth occurred relative to the genotypes home climate.

We estimated fitness changes under future values of MCMT using the norm of reaction generated from the model to predict the growth, mortality, and combined fitness of each genotype at its provenance under historic (1961–1980) and future values of MCMT. Future MCMT values were predicted from an ensemble containing 13 general circulation models (GCMs) from the CMIP6 database, generated from ClimateNA (Wang *et al*., 2016) and available at AdaptWest (AdaptWest Project, 2022). We used the time period of 2041–2070 under the shared socioeconomic pathway 2–4.5, an intermediate scenario in which emissions rise until mid‐century, and then decline (IPCC, 2023). To predict how the relative fitness of *P. balsamifera*, *P. trichocarpa*, and hybrid genotypes may change under future values of MCMT, we predicted which genotypes would have the best performance (highest overall fitness combining growth and survival) across the species ranges and regions with common gardens. For each grid cell, we extracted MCMT values for past and future climates, and identified the genotype with the highest predicted fitness at that temperature.

## Results

### Variation in growth among gardens

Growth increment varied widely among gardens (Fig. S2), with the highest growth occurring at WSU (Washington) and SU (Maryland) gardens, and the lowest growth occurring at the upper and lower winter temperature extremes, particularly at OLLU (Texas), UCM (California), OSU (Oregon), and NDSU (North Dakota). Genotypes typically reached their greatest height gain at intermediate temperatures warmer than their climate of origin, with growth increment decreasing at temperature extremes (Figs S3, S4), suggesting that MCMT exerts selection pressure on poplars, as previously found in high‐latitude tree species (Leites *et al*., 2012, 2019; Yeaman *et al*., 2016; Rehfeldt *et al*., 2018; Mahony *et al*., 2020).

### Factors predicting growth and mortality

**Fig. 2:**
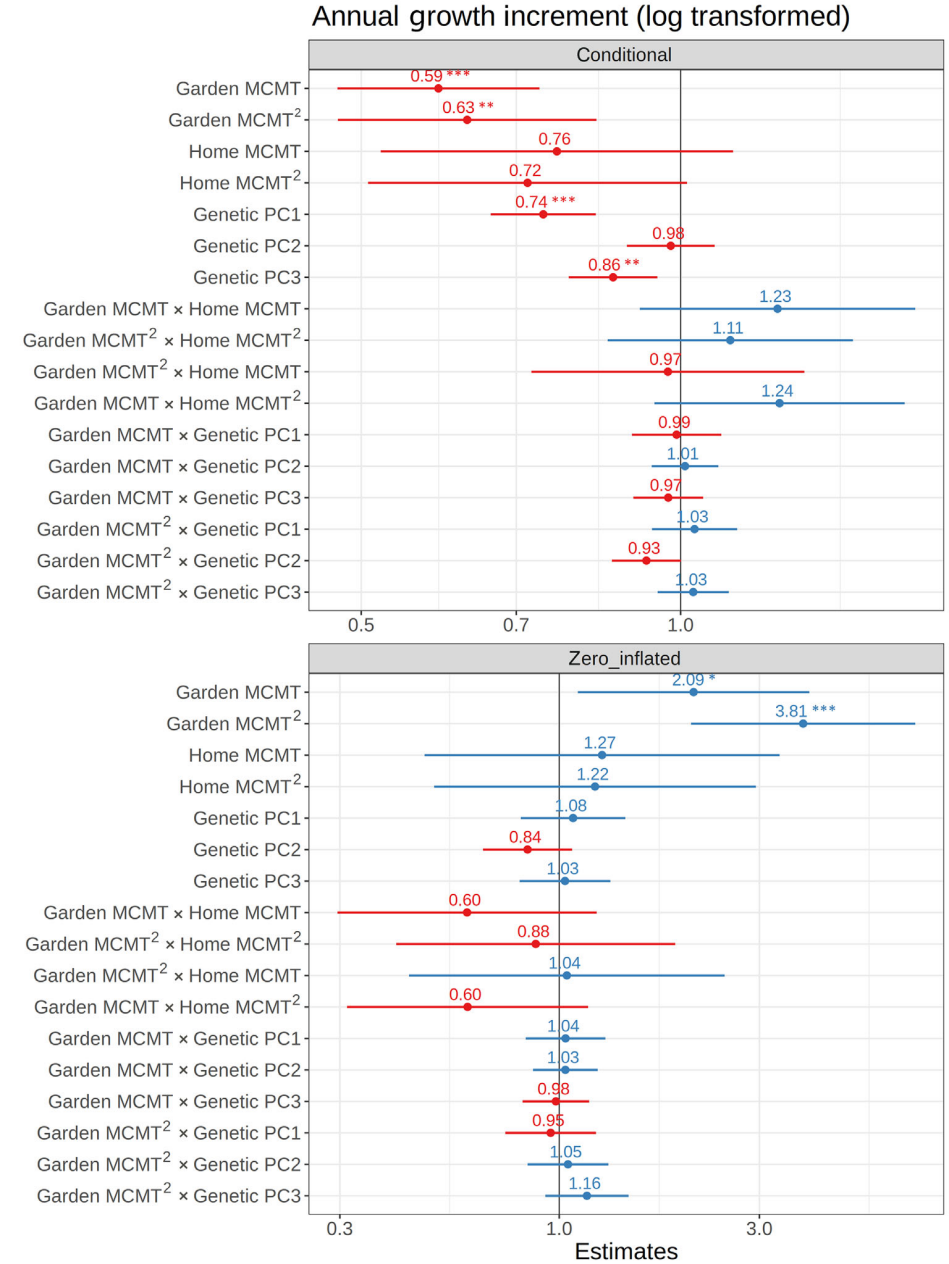
Forest plot of genetic and environmental factors explaining growth and mortality of admixed *Populus trichocarpa* and *Populus balsamifera* genotypes planted in common gardens, which include principal components (PCs) of genetic data, mean coldest month temperature (MCMT) of the garden and home site, and their interactions. Points depict standardized estimates, with an estimate of one indicating no effect, and estimates significantly greater or less than one indicating a positive (blue) and negative (red) effect, respectively. Coefficients are standardized by dividing by their SD to allow comparison of effect sizes among all factors. Lines show the 95% confidence intervals. The top plot shows results for the conditional model (growth increment) and the bottom plot shows the estimates for the zero‐inflated model (mortality); the effects for the two models are reversed because the zero‐inflated model predicts the probability of mortality, which is negatively correlated with growth. *P*‐values were calculated using a type II Wald chi‐squared test, and are indicated as follows: *, *P* < 0.05; **, *P* < 0.01; ***, *P* < 0.001. Plots were created using sjplot (v.2.8.17; Lüdecke, 2024).

We selected MCMT as the climate variable used in each model, because it had the lowest AIC score when predicting growth increment for 2021 (Table S2), and MCMT values in gardens included MCMT values from most historic home environments. We evaluated the effect size and significance of each model factor considering the two model components: the conditional component, representing growth, and the zero‐inflated component, representing the probability of mortality (Fig. 2; Table S3). Here, we report effect sizes as standardized beta coefficients to enable comparison of the relative importance of each factor. Intercepts of random effects are presented in Fig. S5, and effect sizes are in Table S4. The two model components had similar effects, indicating that the same factors associated with increased growth were also associated with low mortality. MCMT of the common garden site and its square term had the greatest effect on growth (slope = −0.53 and −0.46, *P* < 0.001 and = 0.001, respectively; Fig. 2; Table S2). Both had negative effects, indicating decreased growth in climates warmer or colder than the optimum temperature. Intra‐ and interspecific genetic structure represented by PC1 and PC3 both had significant effects on growth, although with a smaller effect size than garden temperature (slope = −0.3, *P* < 0.001 for PC1 and slope = −0.15, *P* = 0.003 for PC3, Table S2). Individuals with greater *P. trichocarpa* ancestry (PC1) and from the three southernmost transects (PC3) had higher growth on average. PC2, which explains genetic structure within *P. trichocarpa* (Fig. S1), was not statistically significant, suggesting that genetic differences between interior and coastal *P. trichocarpa* did not significantly affect growth. Home temperature and its interaction with garden temperature did not significantly affect growth, suggesting that responses to MCMT were better explained by genetic structure than by MCMT of origin. While no genotype × environment interaction terms were significant at the *P* < 0.05 level across both years, Garden MCMT^2^ × Genetic PC2 was significant for a model including only measurements from 2021 (Fig. S6), suggesting a weak effect of variation in the temperature response curve between two *P. trichocarpa* lineages during first‐year growth (Fig. S1). Overall, our results show that, of the factors tested, temperature had the largest effect on growth. Genetics also contributed to variation in growth, which suggests the response to temperature is partially determined by species ancestry and latitudinal variation within both species. Similarly, in the zero‐inflated model, garden MCMT significantly affected the probability of mortality, with increased mortality in sites at the higher and lower temperature extremes. Genetic structure, home MCMT, and their interactions with garden MCMT were not significant, suggesting that among the tested factors, mortality probability was primarily driven by planting site temperature, with limited variation among genotypes.

### Evaluation of model predictive ability

Both conditional (growth) and overall (growth and survival) components of Model 1 generally performed well in predicting the response to climate. The correlation between actual and predicted growth increments when random effects were included in the prediction was 0.795 for the overall model, and 0.809 for the conditional component (Fig. S7). Similarly, the prediction of mortality from the zero‐inflated component was significantly associated with actual mortality (*P* < 0.001; Fig. S8). When random effect estimates from Model 1 were not incorporated, predictive ability decreased to 0.631 and 0.538 for the overall and conditional components, respectively (Fig. S7), indicating that there were garden‐specific effects on plant response not accounted for by garden MCMT, and/or genotype‐specific effects that were not accounted for by home MCMT or the genetic PCs, and year‐specific effects. However, the model consistently underpredicted actual growth increment values, particularly for the tallest trees. For this reason, we focus on the relative differences in growth among genotypes and gardens rather than predicting specific yearly growth increments.

Prediction ability (Pearson's correlation between observed and predicted growth increments) evaluated using leave‐one‐out cross‐validation varied widely among gardens (Fig. S9), with the average being 0.49 for 2021 and 0.38 for 2022, the highest correlation being 0.67 for OSU in 2022 (Oregon), and the lowest correlation being −0.8 for NDSU in 2022 (North Dakota). Most sites with poor predictions were sites with high mortality (NDSU, OLLU, LOCK, UCM, and OSU had > 40% mortality in the first year). Other sites with low prediction ability included SWMN (*r* = 0.058 in 2021 but increasing to 0.47 in 2022) and WI (*r* = 0.397 in 2021, −0.092 in 2022), which are two sites that have low MCMT (Fig. S2) and therefore may provide unique information on responses to lower temperatures. The relationship between predicted probability of mortality and actual mortality was not significant for most gardens and years (Fig. S10), reflecting a low predictive ability for mortality alone.

**Fig. 3:**
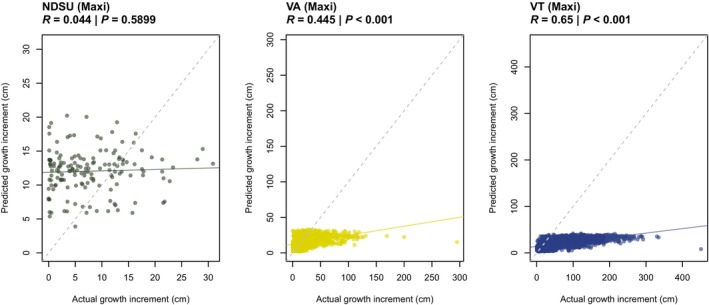
Performance of model predicting yearly growth increment in centimeters (cm) for individual trees belonging to 544 admixed *Populus trichocarpa* and *Populus balsamifera* genotypes (including 500 novel genotypes) and planted in three maxi gardens (including one novel environment, VT). The model was trained on data from 44 genotypes in 17 mini garden environments. Models were evaluated for their predictive ability by comparing the actual growth increment for each tree with that predicted by the conditional model, with no random effects for garden and genotype. *R* values and *P*‐values are given for the Pearson correlation between actual and predicted growth. As the conditional portion of the model predicts growth rather than mortality, individuals that did not survive were removed from the plot and correlation. The dotted gray line indicates the 1 : 1 line, and the solid colored line indicates the best fit.

We also predicted growth for our three ‘maxi’ common garden experiments, which included 500 novel genotypes and one novel site (Fig. 3). Prediction ability was low for NDSU, likely due to high mortality (Pearsons *r* = 0.04 for the conditional model). However, prediction ability for the VA and VT models was relatively high. For the conditional model, the correlation between actual and predicted values for VA was 0.45 and VT 0.65 (Fig. 3), and when dead individuals were included in the overall model VA had a prediction ability of 0.48 and VT 0.53 (Fig. S11). For VA, the predicted probability of mortality was significantly associated with actual mortality (Fig. S12). However, as with the leave‐one‐garden‐out tests, the model underpredicted growth. Taken together, these results suggest that for environments with low mortality, the model can predict relative performance for both novel genotypes and novel environments outside of the training dataset, but is limited in its prediction of specific growth increments.

### The role of genetics in phenotypic prediction across environments

**Fig. 4:**
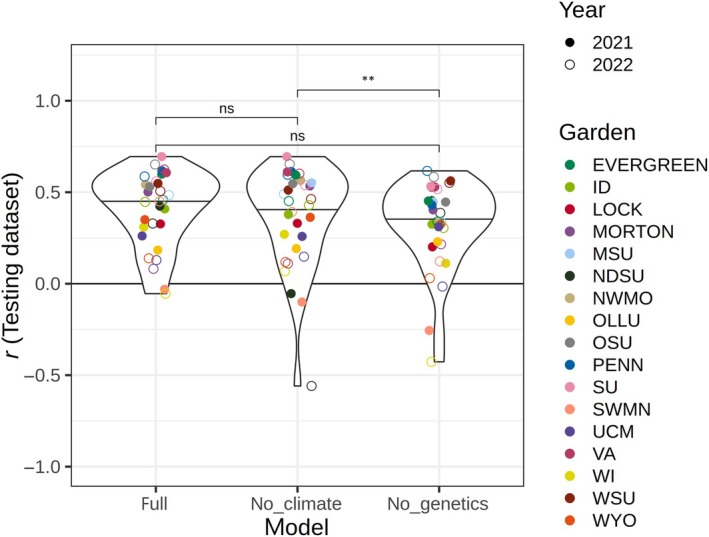
Comparison of performance among models predicting the yearly growth increment for admixed *Populus trichocarpa* and *Populus balsamifera* genotypes planted in common garden environments. Performance was compared among the full model and those excluding provenance climate as represented by mean coldest month temperature (MCMT), or genetic structure represented by genetic PCs (Table 1). Model predictive ability was estimated as the Pearson correlation (*r*) between predicted and observed growth increments for each garden and year predicted using the leave‐one‐out models, in which growth increments were predicted for a single year and garden using a model trained on other gardens. The model including genetic data without home temperature data resulted in better predictive ability than the model with temperature data alone (pairwise Wilcoxon test, *P* = 0.0039). Other comparisons with the overall model and with random effects included are shown in Supporting Information Fig. S13. **, *P* ≤ 0.01; ns, not significant. In violin plots, points show the actual value for each garden and year, jittered horizontally. Violin shapes show frequency distributions, and horizontal lines indicate medians.

When predicting growth and overall performance for each garden and year using leave‐one‐out cross‐validation, the climate‐only model (Model 3) that excluded genetic information had lower performance than the genetics‐only model (Model 2; ANOVA of predictive ability from 17 leave‐one‐out cross‐validation tests, *P* = 0.0039). On average, Model 3 had lower performance than the full model (Model 1), but the two were not significantly different (*P* = 0.061; Table 1; Fig. 4). These results show that genetic information improved predictions when climate data were excluded, but that models excluding either genetics or climate data performed similarly to the full model. When random effects of garden and genotype were included in the predictions, all models performed equally well (Fig. S13). This is likely because all genotypes were included in the training datasets allowing the intercept of each genotype to be estimated, improving predictions of their growth in a novel environment without explicitly including genetic or climate information.

**Fig. 5:**
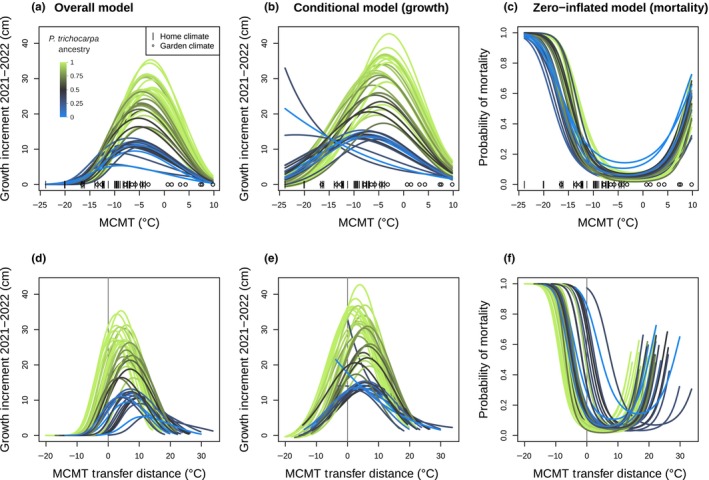
Variation in predicted reaction norms in response to mean coldest month temperature (MCMT) across admixed *Populus* genotypes. The reaction norm of each genotype is shown as a separate line colored by species ancestry, with green indicating *Populus trichocarpa* and blue indicating *Populus balsamifera*. Predictions for the overall model (a, d) incorporate both yearly growth increment in centimeters (cm) and the probability of mortality; predictions for the conditional model (b, e) only predict growth, ignoring the probability of zeros arising from other processes including mortality; and predictions for the zero‐inflated model (c, f) predict the probability of zeros arising from mortality. (a–c) Responses across the range of MCMT values at garden and home climates. Actual values of home climates (|) and garden climates (circle) used for model training are shown on the *x*‐axis. (d, e) Responses based on distance from climate of origin (garden–home MCMT); positive values indicate a warmer climate and negative values indicate a colder climate. If genotypes perform best in environments similar to their home environment, growth should be highest and mortality should be lowest when the transfer distance is 0.

The full model (Model 1) trained on all 17 gardens predicts that genotypes with more *P. trichocarpa* ancestry have higher growth and overall performance in warmer environments (MCMT: *c*. −14°C to 10°C) compared with *P. balsamifera* genotypes, but that genotypes with more *P. balsamifera* ancestry have higher growth in colder climates, consistent with the climatic preferences of the two species (Fig. 5a,b). Biologically unrealistic reaction norms were predicted for two majority‐*P. balsamifera* genotypes, with growth increasing exponentially with decreased winter temperatures (Fig. 5b). These modeled growth responses likely reflect a preference for very cold environments that are not well represented in our gardens, and when the effects of growth and mortality are combined, these genotypes show realistic response curves with optima at relatively low temperatures. Across species ancestries, mortality is predicted to increase at the colder and warmer extremes, with a greater probability of mortality under extreme cold climates compared with warmer climates (Fig. 5c). As with predictions of growth, mortality responses predict that *P. balsamifera* is more cold‐tolerant, with increased probability of mortality in *P. trichocarpa* genotypes occurring at less extreme cold temperatures than for *P. balsamifera* (Fig. 5c). Likewise, the probability of mortality for *P. balsamifera* generally increases with warmer temperatures more than *P. trichocarpa*. Combining the growth and mortality predictions as an overall fitness proxy, genotypes with a majority *P. trichocarpa* ancestry have consistently higher fitness except in the coldest climates, where majority‐ancestry *P. balsamifera* genotypes are predicted to outperform them (Fig. 5a). The overall maximum aboveground growth of *P. balsamifera* genotypes is lower than that of *P. trichocarpa*, which has previously been reported to be a faster‐growing species (Larcheveque *et al*., 2011; Suarez‐Gonzalez *et al*., 2017). Admixed genotypes with majority *P. trichocarpa* ancestry had maximum heights intermediate to the two parental species, while admixed majority *P. balsamifera* genotypes had responses more similar to parental *P. balsamifera* genotypes.

### Rangewide projections of future fitness changes

**Fig. 6:**
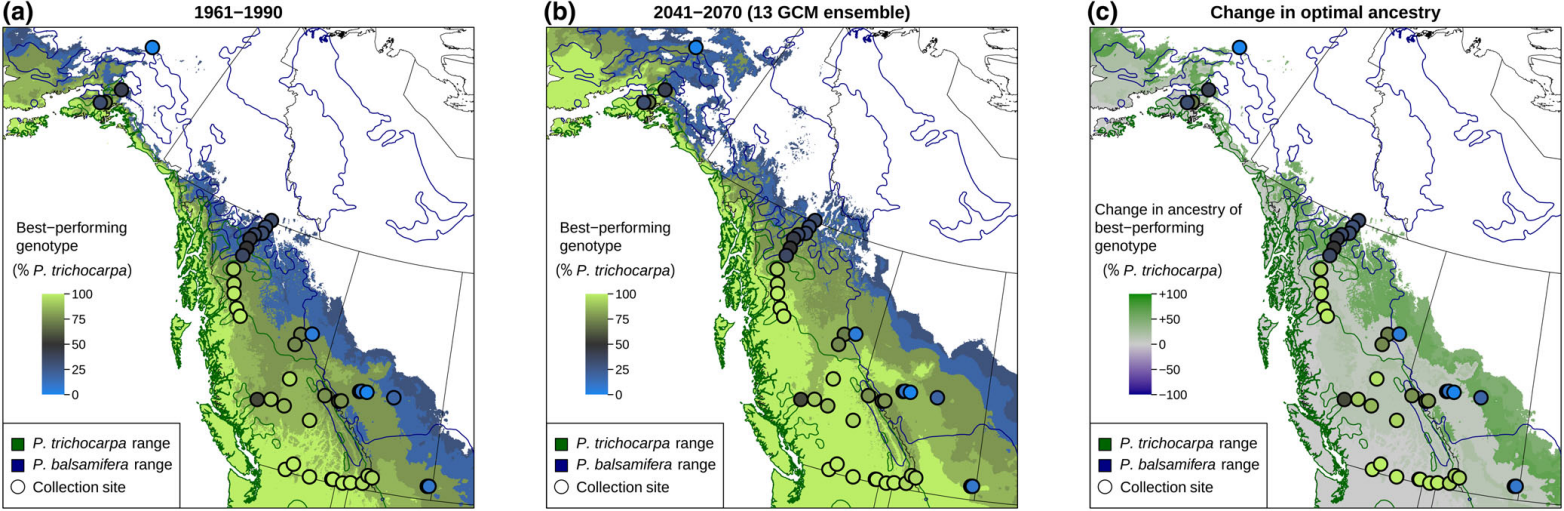
Maps showing the predicted shift in species ancestry in the *Populus* hybrid zone, based on genotype‐specific responses to mean coldest month temperature (MCMT). In (a, b), the color of the base layer shows the species ancestry of the studied genotype which is predicted to have the highest fitness (as measured by growth and mortality) in that location under historic (a) and future climates (b). (c) The change in optimal species ancestry between future (b) and historic (a) climates, indicating regions where increased *Populus trichocarpa* ancestry is expected to be beneficial in green, and regions with no change in gray. As MCMT increases, we predict that genotypes with higher *P. trichocarpa* ancestry may be able to outcompete genotypes with higher *Populus balsamifera* ancestry in some portions of the *P. balsamifera* range, favoring a northeastern shift of the *P. trichocarpa* range and the hybrid zone and into historically colder, more continental regions. Regions with MCMT values outside of the range measured in the common gardens (−13.05°C to 10.85°C) are masked and colored white. Actual ancestry of collected genotypes is shown as circles. Species ranges are shown as dark blue and green outlines (Little, 1971). The same predictions are mapped across North America, including the common garden sites (Supporting Information Fig. S15).

Our full model (Model 1) predicts increased growth and survival under climates with MCMT temperatures warmer than the climate of origin (Figs 5d–f, S14). However, we are limited in our ability to predict responses to multivariate changes in climate. Here, we focus on the relative performance of genotypes rather than absolute changes in performance metrics. Predictions of the best‐performing genotype under historic values of MCMT generally follow previously described species ranges, with *P. trichocarpa* occurring along the west coast of North America and *P. balsamifera* occurring in the more northern and interior regions of the continent (Figs 6, S15). Hybrid genotypes are predicted to outcompete parental species in intermediate regions of the contact zone, particularly in central British Columbia, where sampled genotypes exhibited high levels of admixture. Under future climates (2041–2070 for a ‘middle‐of‐the‐road’ emissions scenario), the model predicts shifts in the best‐performing genotype following the two species' climatic preferences: the regions suitable for *P. trichocarpa* and its backcrosses will shift northward as the climate warms (Fig. 6).

## Discussion

Using 17 common gardens with *Populus* genotypes which originated from a hybrid zone spanning a broad temperature range, we evaluated the genotype‐specific response to warming winter temperatures and predicted future responses across the hybrid zone. We found that: fitness metrics and reaction norms varied among genotypes due to species ancestry and region of origin, consistent with a trade‐off between cold tolerance and growth potential in warmer environments; hybrids displayed reaction norms and temperature optima intermediate between their parental species, suggesting that warming temperatures could favor movement of the hybrid zone; and a significant variance in growth explained by genetic structure illustrates the importance of genetic variation in predicting responses to climate change.

### Question 1: Do genotypes from different climates vary in fitness responses across environments?

Local adaptation to climate can shape not only traits but also their reaction norms across environments, contributing to variation in plastic responses within and across species (Patsiou *et al*., 2020). We found that poplar growth and survival across common garden environments were predicted by species ancestry and by genetic differences associated with a northern and southern region within each species (Fig. 2), illustrating that genetic variation will contribute to spatially varying responses to warming temperatures (Patsiou *et al*., 2020; Martinez del Castillo *et al*., 2024). Performance traits varied along a continuous ancestry gradient between the two parental species, consistent with their climatic niches. In general, individuals with greater *P. trichocarpa* ancestry had greater overall growth, but these genotypes experienced lower growth and increased mortality in cold, continental environments typical of the *P. balsamifera* range (Fig. 5). In addition, genotypes from the three southernmost transects had higher maximum yearly growth than those from the two northern transects (as described by genetic PC3). This is consistent with a trade‐off between growth potential and cold tolerance often observed in temperate or boreal tree species, likely enabling *P. balsamifera* to outcompete *P. trichocarpa* in colder regions (Leites *et al*., 2012; Menon *et al*., 2015; Rehfeldt *et al*., 2018). While no *G* × *E* terms were significant in the model based on 2 yr of data, the effect of garden MCMT^2^ × genetic PC2 and was significant when only measurements from 2021 were included in the model (Fig. S6). This result suggests a weak effect of genetic variation between two *P. trichocarpa* lineages on the plastic response to winter temperatures, which may be partially explained by maternal effects expressed in the first year of growth. Additionally, optimum temperatures varied for each genotype, with *P. trichocarpa* individuals reaching maximum growth in warmer environments than *P. balsamifera* (Fig. 5). This suggests that selection associated with minimum temperatures may be acting to produce different plastic responses across the range of both species and their hybrid zone. While the majority of *P. balsamifera* genotypes had lower overall growth, their climatic range appeared to be wider (Fig. 5), perhaps reflecting adaptation to more continental climates with larger annual temperature ranges (Fig. 1). Generally, the response of hybrid genotypes was intermediate between the parental species; however, some hybrid genotypes have a reduced probability of mortality at lower temperatures (Fig. 5), suggesting that introgression could promote increased cold hardiness (Hamilton *et al*., 2013). However, the effects of species ancestry and geography may be difficult to disentangle, as genotypes from the coldest part of the sampled range (Alaska and northern British Columbia) are admixed (Fig. S1).

In this study, we found that most genotypes reached their fitness optima in environments that were warmer than their climate of origin (Fig. 6), and that genotypes planted at sites having MCMT values similar to their home site did not always perform better than other, nonlocal genotypes (Fig. S16). Taken together, these results suggest genotypes may not be locally adapted to winter temperatures alone when considering either the ‘home vs away’ or ‘local vs foreign’ criteria (Kawecki & Ebert, 2004). This decoupling between the physiological optimum, the climate in which genotypes reach their maximum growth rate, and the ecological optimum, the climate in which they exist in the ecosystem, has been previously observed for temperate tree species (Rehfeldt *et al*., 2018). Higher growth rates in nonlocal environments do not necessarily indicate a lack of local adaptation (Kawecki & Ebert, 2004), but provide important context for predicting responses to climate change, which often assume optimal performance in local climates. Instead, we found that most genotypes, when in gardens with temperatures similar to their climate of origin, were outperformed by a small number of primarily *P. trichocarpa* genotypes. These genotypes may be ideal for planting as part of restoration projects using assisted gene flow or for biofuel production across diverse environments, particularly under increasingly variable and unpredictable climate futures (Sannigrahi *et al*., 2010; Porth & El‐Kassaby, 2015; Mahoney *et al*., 2019). However, if increased growth corresponds to a lack of cold tolerance or lower survival and fecundity (Leites *et al*., 2012, 2019; Yeaman *et al*., 2016; Rehfeldt *et al*., 2018; Mahony *et al*., 2020), caution is warranted when planting in locations susceptible to cold stress. Further work comparing the cold tolerance of these genotypes could determine the lower temperature limits where they are likely to be successful, while reciprocal transplants could be used to test whether local genotypes outperform nonlocal genotypes. Regardless of the mechanisms underlying genetic variation in fitness proxies, the large differences in responses to temperature illustrate the benefit of measuring reaction norms across wide geographic regions within and among species.

Our predictions of increased growth under warming climates for these *Populus* genotypes raise the question: Is active conservation and management of poplar in this region necessary, or should we prioritize species more vulnerable to increased temperatures? Predictions of increased growth should be treated with caution, as they only represent aboveground height and mortality in the first 2 yr of growth and exclude reproductive fitness measures and belowground biomass (Fischer *et al*., 2007). Additionally, we only modeled growth over two growing seasons, in relatively warm climates and with irrigation during the first year, limiting the selective response to cold or drought stresses. In natural environments, major selective weather events may occur only rarely; for example, an unusually cold winter could favor slower growing but cold‐tolerant genotypes over longer timescales than studied here (Rehfeldt *et al*., 2018; Lowry *et al*., 2019). In this case, our dataset may underestimate the benefit that *P. balsamifera* has in colder regions. Similarly, if warming winters are accompanied by summer heat waves, the benefits of warming we observe may not persist long‐term in wild populations. Precipitation levels and timing will also shift with climate change, and these changes can compound with temperature stresses (Arend *et al*., 2013; Gantois, 2022; Zandalinas & Mittler, 2022). However, we were unable to assess the effects of precipitation because most gardens were irrigated in the first year to allow seedling establishment. We also did not consider biotic factors such as competition with more cold‐hardy conifers or pathogen pressure (e.g. *Melampsora* spp.) that may exclude *Populus* from warmer climates (La Mantia *et al*., 2013; McKown *et al*., 2014b; Suarez‐Gonzalez *et al*., 2018a), interactions with belowground communities (Fischer *et al*., 2014), or shifts in herbivore presence. If biotic interactions shift along with climate change, overall fitness could decrease even if higher temperatures favor faster aboveground growth. Lastly, our predictions of the response to climate are limited by the training data used to create the model – in this case, the climatic range of the common gardens (Rogers & Holland, 2021). Because genotypes were largely planted in climates warmer than their climate of origin, most temperature variables (including multivariate PC axes) had little overlap between home and garden climates, limiting our ability to make predictions for growth and mortality in the colder historic climates of origin. We choose to use MCMT as the predictor variable because of its biological importance in past studies (Leites *et al*., 2012, 2019; Yeaman *et al*., 2016; Rehfeldt *et al*., 2018; Mahony *et al*., 2020) and because garden climates included temperatures matching historic home climates for both *P. balsamifera* and *P. trichocarpa* genotypes (Figs 1b,c, 5). Given the limitation of our predictions for the coldest part of the hybrid zone and the potential effects of precipitation, testing how native poplar populations are responding to climate change *in situ* could complement common garden studies (Sharma *et al*., 2022; Astigarraga *et al*., 2024; Martinez del Castillo *et al*., 2024).

### Question 2: How will warming temperatures alter the geography of the two species and their hybrid zone?

Geographic variation in the magnitude of climate change and differences in phenotypic plasticity among genotypes could alter local competition dynamics among genotypes and lead to range shifts for the two species and their hybrid zone. While we predict that local poplar populations across the sampled region could experience increased growth and decreased mortality from warmer winter temperatures, they must contend with competition from nonlocal genotypes that may have greater fitness than local genotypes under novel climates. Species ranges are already beginning to shift in response to climate change (Parmesan, 2006; Bell *et al*., 2014; Astigarraga *et al*., 2024) and multi‐species hybrid zones may also shift (Taylor *et al*., 2014, 2015; Billerman *et al*., 2016; Hamilton & Miller, 2016; Wielstra, 2019; Alexander *et al*., 2022). In this poplar hybrid zone, regions where particular genomic backgrounds dominate may shift under climate change. For example, under higher minimum winter temperatures, *P. balsamifera* may be outcompeted by less cold‐tolerant but faster‐growing genotypes with higher *P. trichocarpa* ancestry (Fig. 6). However, such shifts in genetic composition would rely on gene flow or migration being fast enough to track climate change, either through dispersal of genotypes, seeds or wind‐dispersed pollen (Corlett & Westcott, 2013). While *Populus* can likely disperse over long distances via wind dispersal or river‐dispersed vegetative material (Kling & Ackerly, 2021), geographic barriers to gene flow persist across the hybrid zone, particularly in mountainous regions (Bolte *et al*., 2024). Our model predicts that parental *P. trichocarpa* genotypes may begin to outcompete *P. trichocarpa* backcrosses in central British Columbia, a region with high gene flow (Bolte *et al*., 2024), so this region may see shifts in the genetic composition of poplars (Fig. 6). Conversely, while the regions where *P. trichocarpa* ancestry is favored are predicted to shift to the northeast in Alberta, the Rocky Mountains are a barrier to gene flow that may prevent their dispersal (Bolte *et al*., 2024). Our model also predicts that backcrossed *P. balsamifera* genotypes would perform better than parental *P. balsamifera* in northern, interior North America within the core of the species distribution (Fig. 6). However, the reaction norms of parental and backcrossed *P. balsamifera* are very similar (Fig. 5a) and because we have limited data for parental *P. balsamifera* genotypes and their growth in cold climates, we interpret these as regions where increased *P. balsamifera* ancestry is beneficial, not necessarily that backcrosses will be significantly more successful than parental genotypes.

Spatial changes in the location of the hybrid zone could provide an opportunity for adaptive introgression to occur, enabling individual alleles and novel allelic combinations to track climate change. Ancient and contemporary introgression can allow species to persist as environments change (Kremer & Hipp, 2020; Leroy *et al*., 2020; O'Donnell *et al*., 2021; Buck *et al*., 2023; Yang *et al*., 2023). The presence of admixed genotypes in intermediate climates in this *Populus* hybrid zone and the reaction norms that are intermediate between the two parental species are consistent with the bounded hybrid superiority model, in which hybrids have higher fitness than either parental species in intermediate climates (Hamilton *et al*., 2013; De La Torre *et al*., 2014; Bolte *et al*., 2024). Movement of the *P. trichocarpa* × *P. balsamifera* hybrid zone could allow genes to move across species boundaries at new contact zones, increasing the levels of standing genetic variation in these regions and facilitating introgression of alleles that are beneficial under novel climates.

### Question 3: What information is needed to predict climate change responses in trees?

Like generations of provenance studies, we find that the temperature of the planting site had the greatest effect in determining growth, illustrating the continued value that common garden experiments will have in predicting organisms' responses to climate change (O'Neill *et al*., 2008; Wang *et al*., 2010; Leites *et al*., 2012; Browne *et al*., 2019; Leites & Benito Garzón, 2023; Ye *et al*., 2023). Understanding how growth and survival vary across species, populations, and environments will be essential to predicting shifts in the performance and carbon sequestration abilities of trees under changing climates. We show that a model including genetic PCs but not climate of origin significantly improved predictions of growth compared with a model containing only home and garden climates (Fig. 5), suggesting that universal transfer functions for predicting tree growth (O'Neill *et al*., 2008; Wang *et al*., 2010) could benefit from genetic information, as found by other studies (Mahony *et al*., 2020; Archambeau *et al*., 2022; Putra *et al*., 2023; Li *et al*., 2025). Furthermore, the model that included genetic information but not climate of origin predicted growth equally as well as the full model, suggesting genetic structure may be used to effectively model responses to climate in this system. Genetic isolation‐by‐environment is stronger than isolation‐by‐distance in this system (Bolte *et al*., 2024), suggesting environmental adaptation drives much of the observed genetic structure. Conversely, in species with high gene flow and less structured populations, adaptation to climate may be the primary factor contributing to intraspecific variation, and genetic structure may not be as effective in predicting responses.

### Conclusions

By characterizing genotype‐specific reaction norms across 17 common garden environments, we were able to predict which genotypes should have the highest performance in historic and future climates across the hybrid zone, identifying regions where the contact zone is favored to shift under warming winter temperatures. If migration and gene flow are able to track climate change, a moving hybrid zone could facilitate adaptive introgression of alleles beneficial under novel climates, enabling poplar populations to persist in their ecosystems even as their genetic makeup may change.

## Competing interests

None declared.

## Author contributions

JAH, JH, SK and MCF designed the study and contributed to data analysis and interpretation. AM performed analyses and led data interpretation and writing of the manuscript. AM, JRB‐B, AB, DF, SF, JG, LK, SKK, MWK, JRL, JML, DBL, NM, EM, JPS, KLS, BW and MZ‐P contributed to fieldwork and data collection and provided input on garden‐specific conditions for data analysis and interpretation. All authors contributed to writing the manuscript.

## Disclaimer

The New Phytologist Foundation remains neutral with regard to jurisdictional claims in maps and in any institutional affiliations.

## Supporting information


**Dataset S1** Phenotypic, climatic, and genetic data from individuals in the mini gardens.

**Dataset S2** Phenotypic, climatic, and genetic data from individuals in the maxi gardens.

**Dataset S3** Code and outputs from analysis scripts (html files in Rmarkdown format).

**Fig. S1** Principle components plot based on whole‐genome data for genotypes included in this study.
**Fig. S2** Variation in growth increment across gardens and years.
**Fig. S3** Quadratic response of yearly growth increment to garden MCMT for each year from 2021 to 2023.
**Fig. S4** Quadratic response of 2021 growth increment to garden MCMT and TD, plotted as climate transfer distance.
**Fig. S5** Estimates of the random intercepts of garden sites, block, genotype, and year for Model 1.
**Fig. S6** Comparison of model effects when each year (2021–2023) is analyzed separately.
**Fig. S7** Correlation between actual growth and predicted growth estimated from Model 1 across all 17 common gardens.
**Fig. S8** Relationship between actual survival and the probability of mortality predicted from Model 1 across all 17 common gardens.
**Fig. S9** Correlation between actual growth and predicted growth estimated for each garden and year from leave‐one‐out cross‐validation predictions.
**Fig. S10** Relationship between actual survival and the probability of mortality estimated for each garden and year from leave‐one‐out cross‐validation predictions.
**Fig. S11** Correlation between actual growth and predicted growth estimated for the maxi gardens.
**Fig. S12** Relationship between actual survival and the probability of mortality estimated for the maxi gardens.
**Fig. S13** Comparison of performance among the full model (Model 1) and those excluding provenance climate or genetic structure, shown for four categories of predictions.
**Fig. S14** Predicted increases in mean coldest month temperature (MCMT) and the resulting predicted change in fitness metrics for each genotype at its home site.
**Fig. S15** Maps showing the species ancestry of the studied genotype which is predicted to have highest fitness under historic and future climate across North America, including home and garden sites.
**Fig. S16** Predicted reaction norms plotted separately for each genotype to show their relationship with climate of origin.
**Methods S1** Additional methods with R code for Model 1 and details on model evaluation.
**Table S1** List of common garden sites and abbreviations.
**Table S2** Comparison of Akaike information criterion (AIC) scores across models predicting growth and mortality using different climate variables.
**Table S3** Fixed effects of the Model 1, the linear mixed‐effect model predicting yearly growth increment and mortality probability.
**Table S4** Random effects of Model 1, the linear mixed‐effect model predicting yearly growth increment and mortality probability.
Please note: Wiley is not responsible for the content or functionality of any Supporting Information supplied by the authors. Any queries (other than missing material) should be directed to the *New Phytologist* Central Office.

## Data Availability

Phenotypic data from this study are included as Supporting Information: Dataset S1 contains data from the mini gardens, and Dataset S2 contains data from the maxi gardens. Raw sequencing reads have been archived on NCBI (PRJNA996882). Scripts for filtering genomic data are available at https://github.com/alaynamead/poplar_hybrid_vcf_filtering and are archived on Zenodo (doi: 10.5281/zenodo.17515787). Analysis scripts are available at https://github.com/alaynamead/popup_poplar_reaction_norms and are archived on Zenodo (doi: 10.5281/zenodo.17515155), and corresponding R markdown files with script outputs are provided as Dataset S3.

## References

[nph70787-bib-0001] AdaptWest Project . 2022. *Gridded current and projected climate data for North America at 1km resolution, generated using the ClimateNA v.7.30 software (T. Wang et al*., *2022)* . [WWW document] URL https://adaptwest.databasin.org

[nph70787-bib-0002] Aitken SN , Bemmels JB . 2016. Time to get moving: assisted gene flow of forest trees. Evolutionary Applications 9: 271–290. 27087852 10.1111/eva.12293 PMC4780373

[nph70787-bib-0003] Aitken SN , Whitlock MC . 2013. Assisted gene flow to facilitate local adaptation to climate change. Annual Review of Ecology, Evolution, and Systematics 44: 367–388.

[nph70787-bib-0004] Alexander A , Robbins MB , Holmes J , Moyle RG , Peterson AT . 2022. Limited movement of an avian hybrid zone in relation to regional variation in magnitude of climate change. Molecular Ecology 31: 6634–6648. 36210655 10.1111/mec.16727 PMC9729445

[nph70787-bib-0005] Alexander DH , Novembre J , Lange K . 2009. Fast model‐based estimation of ancestry in unrelated individuals. Genome Research 19: 1655–1664. 19648217 10.1101/gr.094052.109 PMC2752134

[nph70787-bib-0006] Archambeau J , Benito Garzón M , Barraquand F , de Miguel M , Plomion C , González‐Martínez SC . 2022. Combining climatic and genomic data improves range‐wide tree height growth prediction in a forest tree. The American Naturalist 200: E141–E159. 10.1086/720619 36150196

[nph70787-bib-0007] Arend M , Brem A , Kuster TM , Günthardt‐Goerg MS . 2013. Seasonal photosynthetic responses of European oaks to drought and elevated daytime temperature. Plant Biology 15: 169–176. 22776350 10.1111/j.1438-8677.2012.00625.x

[nph70787-bib-0008] Arnold PA , Kruuk LEB , Nicotra AB . 2019. How to analyse plant phenotypic plasticity in response to a changing climate. New Phytologist 222: 1235–1241. 30632169 10.1111/nph.15656

[nph70787-bib-0009] Astigarraga J , Esquivel‐Muelbert A , Ruiz‐Benito P , Rodríguez‐Sánchez F , Zavala MA , Vilà‐Cabrera A , Schelhaas M‐J , Kunstler G , Woodall CW , Cienciala E *et al*. 2024. Relative decline in density of Northern Hemisphere tree species in warm and arid regions of their climate niches. Proceedings of the National Academy of Sciences, USA 121: e2314899121. 10.1073/pnas.2314899121 PMC11252807 38954552

[nph70787-bib-0010] Bell DM , Bradford JB , Lauenroth WK . 2014. Early indicators of change: divergent climate envelopes between tree life stages imply range shifts in the western United States. Global Ecology and Biogeography 23: 168–180.

[nph70787-bib-0011] Billerman SM , Murphy MA , Carling MD . 2016. Changing climate mediates sapsucker (Aves: Sphyrapicus) hybrid zone movement. Ecology and Evolution 6: 7976–7990. 27878070 10.1002/ece3.2507 PMC5108250

[nph70787-bib-0012] Bolte CE , Phannareth T , Zavala‐Paez M , Sutara BN , Can MF , Fitzpatrick MC , Holliday JA , Keller SR , Hamilton JA . 2024. Genomic insights into hybrid zone formation: the role of climate, landscape, and demography in the emergence of a novel hybrid lineage. Molecular Ecology 33: e17430. 38867593 10.1111/mec.17430

[nph70787-bib-0013] Brooks ME , Kristensen K , van Benthem KJ , Magnusson A , Berg CW , Nielsen A , Skaug HJ , Mächler M , Bolker BM . 2017. glmmtmb balances speed and flexibility among packages for zero‐inflated generalized linear mixed modeling. The R Journal 9: 378–400.

[nph70787-bib-0014] Browne L , Wright JW , Fitz‐Gibbon S , Gugger PF , Sork VL . 2019. Adaptational lag to temperature in valley oak (*Quercus lobata*) can be mitigated by genome‐informed assisted gene flow. Proceedings of the National Academy of Sciences, USA 116: 25179–25185. 10.1073/pnas.1908771116 PMC6911187 31767740

[nph70787-bib-0015] Buck R , Ortega‐Del Vecchyo D , Gehring C , Michelson R , Flores‐Rentería D , Klein B , Whipple AV , Flores‐Rentería L . 2023. Sequential hybridization may have facilitated ecological transitions in the Southwestern pinyon pine syngameon. New Phytologist 237: 2435–2449. 36251538 10.1111/nph.18543

[nph70787-bib-0016] Capblancq T , Fitzpatrick MC , Bay RA , Exposito‐Alonso M , Keller SR . 2020. Genomic prediction of (mal)adaptation across current and future climatic landscapes. Annual Review of Ecology, Evolution, and Systematics 51: 245–269.

[nph70787-bib-0017] Chhatre VE , Evans LM , DiFazio SP , Keller SR . 2018. Adaptive introgression and maintenance of a trispecies hybrid complex in range‐edge populations of *Populus* . Molecular Ecology 27: 4820–4838. 30071141 10.1111/mec.14820

[nph70787-bib-0018] Chhetri HB , Macaya‐Sanz D , Kainer D , Biswal AK , Evans LM , Chen J‐G , Collins C , Hunt K , Mohanty SS , Rosenstiel T *et al*. 2019. Multitrait genome‐wide association analysis of *Populus trichocarpa* identifies key polymorphisms controlling morphological and physiological traits. New Phytologist 223: 293–309. 30843213 10.1111/nph.15777

[nph70787-bib-0019] Cooper HF , Best RJ , Andrews LV , Corbin JPM , Garthwaite I , Grady KC , Gehring CA , Hultine KR , Whitham TG , Allan GJ . 2022. Evidence of climate‐driven selection on tree traits and trait plasticity across the climatic range of a riparian foundation species. Molecular Ecology 31: 5024–5040. 35947510 10.1111/mec.16645

[nph70787-bib-0020] Cooper HF , Grady KC , Cowan JA , Best RJ , Allan GJ , Whitham TG . 2019. Genotypic variation in phenological plasticity: reciprocal common gardens reveal adaptive responses to warmer springs but not to fall frost. Global Change Biology 25: 187–200. 30346108 10.1111/gcb.14494

[nph70787-bib-0021] Corlett RT , Westcott DA . 2013. Will plant movements keep up with climate change? Trends in Ecology & Evolution 28: 482–488. 23721732 10.1016/j.tree.2013.04.003

[nph70787-bib-0022] De La Torre AR , Wang T , Jaquish B , Aitken SN . 2014. Adaptation and exogenous selection in a *Picea glauca* × *Picea engelmannii* hybrid zone: implications for forest management under climate change. New Phytologist 201: 687–699. 24200028 10.1111/nph.12540 PMC4285121

[nph70787-bib-0023] Des Marais DL , Hernandez KM , Juenger TE . 2013. Genotype‐by‐environment interaction and plasticity: exploring genomic responses of plants to the abiotic environment. Annual Review of Ecology, Evolution, and Systematics 44: 5–29.

[nph70787-bib-0024] Evans LM , Slavov GT , Rodgers‐Melnick E , Martin J , Ranjan P , Muchero W , Brunner AM , Schackwitz W , Gunter L , Chen J‐G *et al*. 2014. Population genomics of *Populus trichocarpa* identifies signatures of selection and adaptive trait associations. Nature Genetics 46: 1089–1096. 25151358 10.1038/ng.3075

[nph70787-bib-0025] Fischer DG , Chapman SK , Classen AT , Gehring CA , Grady KC , Schweitzer JA , Whitham TG . 2014. Plant genetic effects on soils under climate change. Plant and Soil 379: 1–19.

[nph70787-bib-0026] Fischer DG , Hart SC , LeRoy CJ , Whitham TG . 2007. Variation in below‐ground carbon fluxes along a *Populus* hybridization gradient. New Phytologist 176: 415–425. 17888120 10.1111/j.1469-8137.2007.02167.x

[nph70787-bib-0027] Fitzpatrick MC , Chhatre VE , Soolanayakanahally RY , Keller SR . 2021. Experimental support for genomic prediction of climate maladaptation using the machine learning approach Gradient Forests. Molecular Ecology Resources 21: 2749–2765. 33683822 10.1111/1755-0998.13374

[nph70787-bib-0028] Fitzpatrick MC , Keller SR . 2015. Ecological genomics meets community‐level modelling of biodiversity: mapping the genomic landscape of current and future environmental adaptation. Ecology Letters 18: 1–16. 25270536 10.1111/ele.12376

[nph70787-bib-0029] Gantois J . 2022. New tree‐level temperature response curves document sensitivity of tree growth to high temperatures across a US‐wide climatic gradient. Global Change Biology 28: 6002–6020. 35733243 10.1111/gcb.16313

[nph70787-bib-0030] Geraldes A , Farzaneh N , Grassa CJ , McKown AD , Guy RD , Mansfield SD , Douglas CJ , Cronk QCB . 2014. Landscape genomics of *Populus trichocarpa*: the role of hybridization, limited gene flow, and natural selection in shaping patterns of population structure. Evolution 68: 3260–3280. 25065449 10.1111/evo.12497

[nph70787-bib-0031] Grady KC , Kolb TE , Ikeda DH , Whitham TG . 2015. A bridge too far: cold and pathogen constraints to assisted migration of riparian forests. Restoration Ecology 23: 811–820.

[nph70787-bib-0032] Gray LK , Gylander T , Mbogga MS , Chen P , Hamann A . 2011. Assisted migration to address climate change: recommendations for aspen reforestation in western Canada. Ecological Applications 21: 1591–1603. 21830704 10.1890/10-1054.1

[nph70787-bib-0033] Hamilton JA , Lexer C , Aitken SN . 2013. Genomic and phenotypic architecture of a spruce hybrid zone (*Picea sitchensis* × *P. glauca*). Molecular Ecology 22: 827–841. 22967172 10.1111/mec.12007

[nph70787-bib-0034] Hamilton JA , Miller JM . 2016. Adaptive introgression as a resource for management and genetic conservation in a changing climate. Conservation Biology 30: 33–41. 26096581 10.1111/cobi.12574

[nph70787-bib-0035] Hijmans RJ . 2025. terra: spatial data analysis . [WWW document] URL https://rspatial.github.io/terra/index.html [accessed 24 September 2025].

[nph70787-bib-0036] Holliday JA , Zhou L , Bawa R , Zhang M , Oubida RW . 2016. Evidence for extensive parallelism but divergent genomic architecture of adaptation along altitudinal and latitudinal gradients in *Populus trichocarpa* . New Phytologist 209: 1240–1251. 26372471 10.1111/nph.13643

[nph70787-bib-0037] Hord AM , Fischer DG , Schweitzer JA , LeRoy CJ , Whitham TG , Bailey JK . 2025. Hybrid introgression as a mechanism of rapid evolution and resilience to climate change in a riparian tree species. Communications Biology 8: 1173. 40775051 10.1038/s42003-025-08410-3 PMC12331986

[nph70787-bib-0038] Hu M‐C , Pavlicova M , Nunes EV . 2011. Zero‐inflated and hurdle models of count data with extra zeros: examples from an HIV‐risk reduction intervention trial. The American Journal of Drug and Alcohol Abuse 37: 367–375. 21854279 10.3109/00952990.2011.597280 PMC3238139

[nph70787-bib-0039] Ikeda DH , Max TL , Allan GJ , Lau MK , Shuster SM , Whitham TG . 2017. Genetically informed ecological niche models improve climate change predictions. Global Change Biology 23: 164–176. 27543682 10.1111/gcb.13470

[nph70787-bib-0040] IPCC . 2023. Climate change 2023: synthesis report. Contribution of Working Groups I, II and III to the Sixth Assessment Report of the Intergovernmental Panel on Climate Change . doi: 10.59327/IPCC/AR6-978929169164.

[nph70787-bib-0041] Janes JK , Hamilton JA . 2017. Mixing it up: the role of hybridization in forest management and conservation under climate change. Forests 8: 237.

[nph70787-bib-0042] Jansson S , Douglas CJ . 2007. *Populus*: a model system for plant biology. Annual Review of Plant Biology 58: 435–458. 10.1146/annurev.arplant.58.032806.103956 17280524

[nph70787-bib-0043] Kawecki TJ , Ebert D . 2004. Conceptual issues in local adaptation. Ecology Letters 7: 1225–1241.

[nph70787-bib-0044] Keller SR , Levsen N , Olson MS , Tiffin P . 2012. Local adaptation in the flowering‐time gene network of balsam poplar, *Populus balsamifera* L. Molecular Biology and Evolution 29: 3143–3152. 22513286 10.1093/molbev/mss121

[nph70787-bib-0045] Keller SR , Olson MS , Silim S , Schroeder W , Tiffin P . 2010. Genomic diversity, population structure, and migration following rapid range expansion in the balsam poplar, *Populus balsamifera* . Molecular Ecology 19: 1212–1226. 20163548 10.1111/j.1365-294X.2010.04546.x

[nph70787-bib-0046] Keller SR , Soolanayakanahally RY , Guy RD , Silim SN , Olson MS , Tiffin P . 2011. Climate‐driven local adaptation of ecophysiology and phenology in balsam poplar, *Populus balsamifera* L. (Salicaceae). American Journal of Botany 98: 99–108. 21613088 10.3732/ajb.1000317

[nph70787-bib-0047] Kling MM , Ackerly DD . 2021. Global wind patterns shape genetic differentiation, asymmetric gene flow, and genetic diversity in trees. Proceedings of the National Academy of Sciences, USA 118: e2017317118. 10.1073/pnas.2017317118 PMC8092467 33875589

[nph70787-bib-0048] Kremer A , Hipp AL . 2020. Oaks: an evolutionary success story. New Phytologist 226: 987–1011. 31630400 10.1111/nph.16274 PMC7166131

[nph70787-bib-0049] La Mantia J , Klápště J , El‐Kassaby YA , Azam S , Guy RD , Douglas CJ , Mansfield SD , Hamelin R . 2013. Association analysis identifies *Melampsora* × *columbiana* poplar leaf rust resistance SNPs. PLoS ONE 8: e78423. 24236018 10.1371/journal.pone.0078423 PMC3827267

[nph70787-bib-0099] Larcheveque M , Maurel M , Desrochers A , Larocque GR . 2011. How does drought tolerance compare between two improved hybrids of balsam poplar and an unimproved native species? Tree Physiology 31: 240–249. 21444373 10.1093/treephys/tpr011

[nph70787-bib-0050] Leites L , Benito Garzón M . 2023. Forest tree species adaptation to climate across biomes: building on the legacy of ecological genetics to anticipate responses to climate change. Global Change Biology 29: 4711–4730. 37029765 10.1111/gcb.16711

[nph70787-bib-0051] Leites LP , Rehfeldt GE , Steiner KC . 2019. Adaptation to climate in five eastern North America broadleaf deciduous species: growth clines and evidence of the growth‐cold tolerance trade‐off. Perspectives in Plant Ecology, Evolution and Systematics 37: 64–72.

[nph70787-bib-0052] Leites LP , Robinson AP , Rehfeldt GE , Marshall JD , Crookston NL . 2012. Height‐growth response to climatic changes differs among populations of Douglas‐fir: a novel analysis of historic data. Ecological Applications 22: 154–165. 22471081 10.1890/11-0150.1

[nph70787-bib-0053] Leroy T , Rougemont Q , Dupouey J‐L , Bodénès C , Lalanne C , Belser C , Labadie K , Le Provost G , Aury J‐M , Kremer A *et al*. 2020. Massive postglacial gene flow between European white oaks uncovered genes underlying species barriers. New Phytologist 226: 1183–1197. 31264219 10.1111/nph.16039 PMC7166129

[nph70787-bib-0054] Li F , Gates DJ , Buckler ES , Hufford MB , Janzen GM , Rellán‐Álvarez R , Rodríguez‐Zapata F , Navarro JAR , Sawers RJH , Snodgrass SJ *et al*. 2025. Environmental data provide marginal benefit for predicting climate adaptation. PLoS Genetics 21: e1011714. 40489511 10.1371/journal.pgen.1011714 PMC12173371

[nph70787-bib-0055] Little EL . 1971. Atlas of United States trees. Washington, DC, USA: U.S. Department of Agriculture, Forest Service.

[nph70787-bib-0056] Love SJ , Schweitzer JA , Woolbright SA , Bailey JK . 2023. Sky islands are a global tool for predicting the ecological and evolutionary consequences of climate change. Annual Review of Ecology, Evolution, and Systematics 54: 219–236.

[nph70787-bib-0057] Lowry DB , Lovell JT , Zhang L , Bonnette J , Fay PA , Mitchell RB , Lloyd‐Reilley J , Boe AR , Wu Y , Rouquette FM *et al*. 2019. QTL × environment interactions underlie adaptive divergence in switchgrass across a large latitudinal gradient. Proceedings of the National Academy of Sciences, USA 116: 12933–12941. 10.1073/pnas.1821543116 PMC6600931 31182579

[nph70787-bib-0058] Lüdecke D . 2024. sjplot: data visualization for statistics in social science . [WWW document] URL https://strengejacke.github.io/sjPlot/ [accessed 23 September 2025].

[nph70787-bib-0059] Mahoney SM , Mike JB , Parker JM , Lassiter LS , Whitham TG . 2019. Selection for genetics‐based architecture traits in a native cottonwood negatively affects invasive tamarisk in a restoration field trial. Restoration Ecology 27: 15–22.

[nph70787-bib-0060] Mahony CR , MacLachlan IR , Lind BM , Yoder JB , Wang T , Aitken SN . 2020. Evaluating genomic data for management of local adaptation in a changing climate: a lodgepole pine case study. Evolutionary Applications 13: 116–131. 31892947 10.1111/eva.12871 PMC6935591

[nph70787-bib-0061] Martinez del Castillo E , Torbenson MCA , Reinig F , Tejedor E , de Luis M , Esper J . 2024. Contrasting future growth of Norway spruce and scots pine forests under warming climate. Global Change Biology 30: e17580. 39548695 10.1111/gcb.17580

[nph70787-bib-0062] McKown AD , Guy RD , Klápště J , Geraldes A , Friedmann M , Cronk QCB , El‐Kassaby YA , Mansfield SD , Douglas CJ . 2014a. Geographical and environmental gradients shape phenotypic trait variation and genetic structure in *Populus trichocarpa* . New Phytologist 201: 1263–1276. 24491114 10.1111/nph.12601

[nph70787-bib-0063] McKown AD , Guy RD , Quamme L , Klápště J , Mantia JL , Constabel CP , El‐Kassaby YA , Hamelin RC , Zifkin M , Azam MS . 2014b. Association genetics, geography and ecophysiology link stomatal patterning in *Populus trichocarpa* with carbon gain and disease resistance trade‐offs. Molecular Ecology 23: 5771–5790. 25319679 10.1111/mec.12969

[nph70787-bib-0064] McKown AD , Klápště J , Guy RD , Geraldes A , Porth I , Hannemann J , Friedmann M , Muchero W , Tuskan GA , Ehlting J *et al*. 2014c. Genome‐wide association implicates numerous genes underlying ecological trait variation in natural populations of *Populus trichocarpa* . New Phytologist 203: 535–553. 24750093 10.1111/nph.12815

[nph70787-bib-0065] Menon M , Barnes WJ , Olson MS . 2015. Population genetics of freeze tolerance among natural populations of *Populus balsamifera* across the growing season. New Phytologist 207: 710–722. 25809016 10.1111/nph.13381

[nph70787-bib-0066] O'Donnell ST , Fitz‐Gibbon ST , Sork VL . 2021. Ancient introgression between distantly related white oaks (*Quercus* sect. *Quercus*) shows evidence of climate‐associated asymmetric gene exchange. Journal of Heredity 112: 663–670. 34508641 10.1093/jhered/esab053

[nph70787-bib-0067] Oksanen J , Simpson GL , Blanchet FG , Kindt R , Legendre P , Minchin PR , O'Hara RB , Solymos P , Stevens MHH , Szoecs E *et al*. 2024. vegan: community ecology package . [WWW document] URL https://vegandevs.github.io/vegan/ [accessed 23 September 2025].

[nph70787-bib-0068] O'Neill GA , Hamann A , Wang T . 2008. Accounting for population variation improves estimates of the impact of climate change on species’ growth and distribution. Journal of Applied Ecology 45: 1040–1049.

[nph70787-bib-0069] Parmesan C . 2006. Ecological and evolutionary responses to recent climate change. Annual Review of Ecology, Evolution, and Systematics 37: 637–669.

[nph70787-bib-0070] Patsiou TS , Shestakova TA , Klein T , di Matteo G , Sbay H , Chambel MR , Zas R , Voltas J . 2020. Intraspecific responses to climate reveal nonintuitive warming impacts on a widespread thermophilic conifer. New Phytologist 228: 525–540. 32402106 10.1111/nph.16656

[nph70787-bib-0071] Porth I , El‐Kassaby YA . 2015. Using *Populus* as a lignocellulosic feedstock for bioethanol. Biotechnology Journal 10: 510–524. 25676392 10.1002/biot.201400194

[nph70787-bib-0072] Putra AR , Yen JDL , Fournier‐Level A . 2023. Forecasting trait responses in novel environments to aid seed provenancing under climate change. Molecular Ecology Resources 23: 565–580. 36308465 10.1111/1755-0998.13728

[nph70787-bib-0073] R Core Team . 2024. R: a language and environment for statistical computing. Vienna, Austria: R Foundation for Statistical Computing.

[nph70787-bib-0074] Rehfeldt GE , Leites LP , Joyce DG , Weiskittel AR . 2018. Role of population genetics in guiding ecological responses to climate. Global Change Biology 24: 858–868. 28862811 10.1111/gcb.13883

[nph70787-bib-0075] Rogers AR , Holland JB . 2021. Environment‐specific genomic prediction ability in maize using environmental covariates depends on environmental similarity to training data. G3: Genes, Genomes, Genetics 12: jkab440. 10.1093/g3journal/jkab440 PMC9245610 35100364

[nph70787-bib-0076] Sannigrahi P , Ragauskas AJ , Tuskan GA . 2010. Poplar as a feedstock for biofuels: a review of compositional characteristics. Biofuels, Bioproducts and Biorefining 4: 209–226.

[nph70787-bib-0077] Sharma S , Andrus R , Bergeron Y , Bogdziewicz M , Bragg DC , Brockway D , Cleavitt NL , Courbaud B , Das AJ , Dietze M *et al*. 2022. North American tree migration paced by climate in the West, lagging in the East. Proceedings of the National Academy of Sciences, USA 119: e2116691118. 10.1073/pnas.2116691118 PMC8784119 34983867

[nph70787-bib-0078] Slavov GT , DiFazio SP , Martin J , Schackwitz W , Muchero W , Rodgers‐Melnick E , Lipphardt MF , Pennacchio CP , Hellsten U , Pennacchio LA *et al*. 2012. Genome resequencing reveals multiscale geographic structure and extensive linkage disequilibrium in the forest tree *Populus trichocarpa* . New Phytologist 196: 713–725. 22861491 10.1111/j.1469-8137.2012.04258.x

[nph70787-bib-0079] Suarez‐Gonzalez A , Hefer CA , Christe C , Corea O , Lexer C , Cronk QCB , Douglas CJ . 2016. Genomic and functional approaches reveal a case of adaptive introgression from *Populus balsamifera* (balsam poplar) in *P. trichocarpa* (black cottonwood). Molecular Ecology 25: 2427–2442. 26825293 10.1111/mec.13539

[nph70787-bib-0080] Suarez‐Gonzalez A , Hefer CA , Lexer C , Cronk QCB , Douglas CJ . 2018a. Scale and direction of adaptive introgression between black cottonwood (*Populus trichocarpa*) and balsam poplar (*P. balsamifera*). Molecular Ecology 27: 1667–1680. 29575353 10.1111/mec.14561

[nph70787-bib-0098] Suarez‐Gonzalez A , Hefer CA , Lexer C , Douglas CJ , Cronk QCB . 2017. Introgression from *Populus balsamifera* underlies adaptively significant variation and range boundaries in *P. trichocarpa* . New Phytologist 217: 416–427. 29124769 10.1111/nph.14779

[nph70787-bib-0081] Suarez‐Gonzalez A , Lexer C , Cronk QCB . 2018b. Adaptive introgression: a plant perspective. Biology Letters 14: 20170688. 29540564 10.1098/rsbl.2017.0688 PMC5897607

[nph70787-bib-0082] Taylor SA , Larson EL , Harrison RG . 2015. Hybrid zones: windows on climate change. Trends in Ecology & Evolution 30: 398–406. 25982153 10.1016/j.tree.2015.04.010 PMC4794265

[nph70787-bib-0083] Taylor SA , White TA , Hochachka WM , Ferretti V , Curry RL , Lovette I . 2014. Climate‐mediated movement of an avian hybrid zone. Current Biology 24: 671–676. 24613306 10.1016/j.cub.2014.01.069

[nph70787-bib-0084] Van Nuland ME , Vincent JB , Ware IM , Mueller LO , Bayliss SLJ , Beals KK , Schweitzer JA , Bailey JK . 2020. Intraspecific trait variation across elevation predicts a widespread tree species' climate niche and range limits. Ecology and Evolution 10: 3856–3867. 32489616 10.1002/ece3.5969 PMC7244802

[nph70787-bib-0085] VanWallendael A , Lowry DB , Hamilton JA . 2022. One hundred years into the study of ecotypes, new advances are being made through large‐scale field experiments in perennial plant systems. Current Opinion in Plant Biology 66: 102152. 35065527 10.1016/j.pbi.2021.102152

[nph70787-bib-0086] Via S , Gomulkiewicz R , De Jong G , Scheiner SM , Schlichting CD , Tienderen PHV . 1995. Adaptive phenotypic plasticity: consensus and controversy. Trends in Ecology & Evolution 10: 212–217. 21237012 10.1016/s0169-5347(00)89061-8

[nph70787-bib-0087] Wang T , Hamann A , Spittlehouse D , Carroll C . 2016. Locally downscaled and spatially customizable climate data for historical and future periods for North America. PLoS ONE 11: e0156720. 27275583 10.1371/journal.pone.0156720 PMC4898765

[nph70787-bib-0088] Wang T , Hamann A , Yanchuk A , O'Neill GA , Aitken SN . 2006. Use of response functions in selecting lodgepole pine populations for future climates. Global Change Biology 12: 2404–2416.

[nph70787-bib-0089] Wang T , O'Neill GA , Aitken SN . 2010. Integrating environmental and genetic effects to predict responses of tree populations to climate. Ecological Applications 20: 153–163. 20349837 10.1890/08-2257.1

[nph70787-bib-0090] Wielstra B . 2019. Historical hybrid zone movement: more pervasive than appreciated. Journal of Biogeography 46: 1300–1305.

[nph70787-bib-0091] Woolbright SA , Whitham TG , Gehring CA , Allan GJ , Bailey JK . 2014. Climate relicts and their associated communities as natural ecology and evolution laboratories. Trends in Ecology & Evolution 29: 406–416. 24932850 10.1016/j.tree.2014.05.003

[nph70787-bib-0092] Yang N , Wang Y , Liu X , Jin M , Vallebueno‐Estrada M , Calfee E , Chen L , Dilkes BP , Gui S , Fan X *et al*. 2023. Two teosintes made modern maize. Science 382: eadg8940. 38033071 10.1126/science.adg8940

[nph70787-bib-0093] Ye Z , O'Neill GA , Wang T . 2023. Optimization and validation of universal response functions for interior spruce (*Picea glauca*, *Picea engelmannii*, and their hybrids). Forest Ecology and Management 550: 121509.

[nph70787-bib-0094] Yeaman S , Hodgins KA , Lotterhos KE , Suren H , Nadeau S , Degner JC , Nurkowski KA , Smets P , Wang T , Gray LK *et al*. 2016. Convergent local adaptation to climate in distantly related conifers. Science 353: 1431–1433. 27708038 10.1126/science.aaf7812

[nph70787-bib-0095] Zandalinas SI , Mittler R . 2022. Plant responses to multifactorial stress combination. New Phytologist 234: 1161–1167. 35278228 10.1111/nph.18087

[nph70787-bib-0096] Zhang M , Suren H , Holliday JA . 2019. Phenotypic and genomic local adaptation across latitude and altitude in *Populus trichocarpa* . Genome Biology and Evolution 11: 2256–2272. 31298685 10.1093/gbe/evz151 PMC6735766

[nph70787-bib-0097] Zhou L , Bawa R , Holliday JA . 2014. Exome resequencing reveals signatures of demographic and adaptive processes across the genome and range of black cottonwood (*Populus trichocarpa*). Molecular Ecology 23: 2486–2499. 24750333 10.1111/mec.12752

