# Supplementary material for: Variation in responses to temperature in admixed Populus genotypes predicts geographic shifts in regions where hybrids are favored
Source: New Phytol. 2025 Nov 30;249(3):1509–26. doi: 10.1111/nph.70787 (PMC12780328; doi:10.1111/nph.70787)
Supplement: Supplementary file 4 — Fig. S1 Principle components plot based on whole‐genome data for genotypes included in this study. Fig. S2 Variation in growth increment across gardens and years. Fig. S3 Quadratic response of yearly growth increment to garden MCMT for each year from 2021 to 2023. Fig. S4 Quadratic response of 2021 growth increment to garden MCMT and TD, plotted as climate transfer distance. Fig. S5 Estimates of the random intercepts of garden sites, block, genotype, and year for Model 1. Fig. S6 Comparison of model effects when each year (2021–2023) is analyzed separately. Fig. S7 Correlation between actual growth and predicted growth estimated from Model 1 across all 17 common gardens. Fig. S8 Relationship between actual survival and the probability of mortality predicted from Model 1 across all 17 common gardens. Fig. S9 Correlation between actual growth and predicted growth estimated for each garden and year from leave‐one‐out cross‐validation predictions. Fig. S10 Relationship between actual survival and the probability of mortality estimated for each garden and year from leave‐one‐out cross‐validation predictions. Fig. S11 Correlation between actual growth and predicted growth estimated for the maxi gardens. Fig. S12 Relationship between actual survival and the probability of mortality estimated for the maxi gardens. Fig. S13 Comparison of performance among the full model (Model 1) and those excluding provenance climate or genetic structure, shown for four categories of predictions. Fig. S14 Predicted increases in mean coldest month temperature (MCMT) and the resulting predicted change in fitness metrics for each genotype at its home site. Fig. S15 Maps showing the species ancestry of the studied genotype which is predicted to have highest fitness under historic and future climate across North America, including home and garden sites. Fig. S16 Predicted reaction norms plotted separately for each genotype to show their relationship with climate of origin. Methods S1 Additional [file NPH-249-1509-s001.pdf]

New Phytologist Supporting Information

Article title: Variation in responses to temperature in admixed *Populus* genotypes predicts geographic shifts in regions where hybrids are favored

Authors: Alayna Mead*, Joie R. Beasley-Bennett, Andrew Bleich, Dylan Fischer, Shelby Flint, Julie Golightly, Lee Kalcsits, Sara K. Klopf, Mason W. Kulbaba, Jesse R. Lasky, Jared M. LeBoldus, David B. Lowry, Nora Mitchell, Emily Moran, Jason P. Sexton, Kelsey L. Søndreli, Baxter Worthing, Michelle Zavala-Paez, Matthew C. Fitzpatrick, Jason Holliday, Stephen Keller, Jill A. Hamilton

*Corresponding author: alaynamead@psu.edu

Article acceptance date: 21 October 2025

Methods S1: Additional methods with R code for Model 1 and details on model evaluation.

Figure S1: Principle components plot based on whole-genome data for genotypes included in this study.

Figure S2: Variation in growth increment across gardens and years.

Figure S3: Quadratic response of yearly growth increment to garden MCMT for each year from 2021-2023.

Figure S4: Quadratic response of 2021 growth increment to garden MCMT and TD, plotted as climate transfer distance.

Figure S5: Estimates of the random intercepts of garden sites, block, genotype, and year for Model 1.

Figure S6: Comparison of model effects when each year (2021-2023) is analyzed separately.

Figure S7: Correlation between actual growth and predicted growth estimated from Model 1 across all 17 common gardens.

Figure S8: Relationship between actual survival and the probability of mortality predicted from Model 1 across all 17 common gardens.

Figure S9: Correlation between actual growth and predicted growth estimated for each garden and year from leave-one-out cross validation predictions.

Figure S10: Relationship between actual survival and the probability of mortality estimated for each garden and year from leave-one-out cross validation predictions.

Figure S11: Correlation between actual growth and predicted growth estimated for the maxi gardens.

Figure S12: Relationship between actual survival and the probability of mortality estimated for the maxi gardens.

Figure S13: Comparison of performance among the full model (Model 1) and those excluding provenance climate or genetic structure, shown for four categories of predictions.

Figure S14: Predicted increases in mean coldest month temperature (MCMT) and the resulting predicted change in fitness metrics for each genotype at its home site.

Figure S15: Maps showing the species ancestry of the studied genotype which is predicted to have highest fitness under historic and future climate across North America, including home and garden sites.

Figure S16: Predicted reaction norms plotted separately for each genotype to show their relationship with climate of origin.

Table S1: List of common garden sites and abbreviations.

Table S2: Comparison of Akaike information criterion (AIC) scores across models predicting growth and mortality using different climate variables.

Table S3: Fixed effects of the Model 1, the linear mixed-effect model predicting yearly growth increment and mortality probability.

Table S4: Random effects of Model 1, the linear mixed-effect model predicting yearly growth increment and mortality probability.

Methods S1

Model function

We implemented Model 1 (main text) using the glmmTMB function from the glmmTMB package version 1.1.11 (Brooks *et al.*, 2017) in R version 4.5.1 (R Core Team, 2024) using the following formula:

log(growth increment + 1) ~ garden MCMT × home MCMT + garden $MCMT^2$ × home $MCMT^2$ + garden $MCMT^2$ × home MCMT + garden MCMT × home $MCMT^2$ + genetic PC1 × garden MCMT + genetic PC2 × garden MCMT + genetic PC3 × garden MCMT + genetic PC1 × garden $MCMT^2$ + genetic PC2 × garden $MCMT^2$ + genetic PC3 × garden $MCMT^2$ + (1 | genotype) + (1 | garden/block) + (1 | year) + (1 | individual)

We used a gaussian model for the conditional component (family = gaussian()) and used the same formula for the zero-inflated component of the model (ziformula = ~.). For complete code used, see the script "transfer_function_multiyear_linear_mixed_effects_model.Rmd."

Model evaluation

We predicted heights for individuals based on the full model using the predict function from the glmmTMB package and calculated the Pearson correlation between the actual heights and the predicted heights. To quantify the model's predictive ability for each year, we calculated the Pearson correlation between the actual heights and the predicted heights (conditional component) as well as the predicted heights when the probability of mortality was included (overall model). To calculate predictive ability for the conditional component, we removed the individuals with a measured height of zero (which is accounted for in the zero-inflated component of the model). We also evaluated model predictive ability with and without the random effects of garden, block, and genotype. Including random effects (using option re.form=NULL in the predict function) accounts for the varying intercepts associated with each group, while excluding them and setting all random effects to zero (re.form = NA) makes predictions based on the fixed effects of climate and genetics only. When the model was used to predict growth in genotypes or gardens not included in the training set, a new random effect was predicted.

We also quantified the predictive ability for the zero-inflated model component estimating mortality rate by testing for a significant relationship between the probability of mortality predicted by the model, and the actual mortality, measured as a binary (yes/no) variable. We fit a generalized linear model with a binomial link function in R using the glm function. We evaluated the model fit using the p-value; however, R^2 values are not estimated for GLMs and are not reported here. Instead, we focus on the predictive ability of the overall model, which incorporates both the conditional and zero-inflated components of the model.

References

Brooks ME, Kristensen K, Benthem KJ van, Magnusson A, Berg CW, Nielsen A, Skaug HJ, Mächler M, Bolker BM. **2017**. glmmTMB Balances Speed and Flexibility Among Packages for Zero-inflated Generalized Linear Mixed Modeling. *The R Journal* **9**: 378–400.

R Core Team. **2024**. R: A Language and Environment for Statistical Computing.

Supplementary Figures

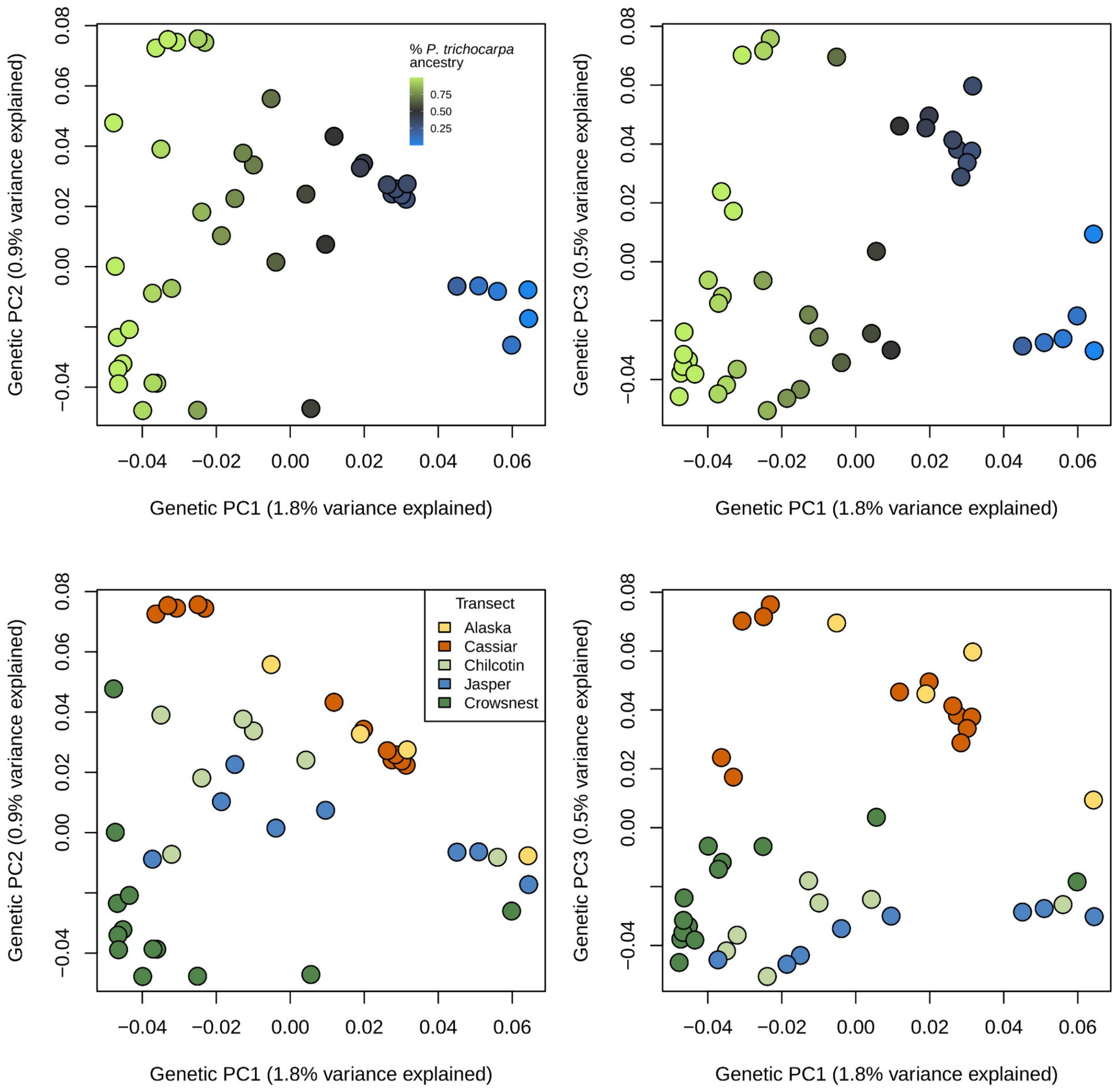

Figure S1. Genomic principle components 1-3 for each genotype, which were used to estimate the effect of genetic structure on phenotypic responses to climate. Top: colors represent species ancestry estimated from ADMIXTURE at K=2, with green representing *Populus trichocarpa* ancestry and blue representing *P. balsamifera* ancestry. Bottom: colors represent transect where genotypes were collected, as described in Bolte et al., (2024) listed from northernmost (Alaska) to southernmost (Crowsnest).

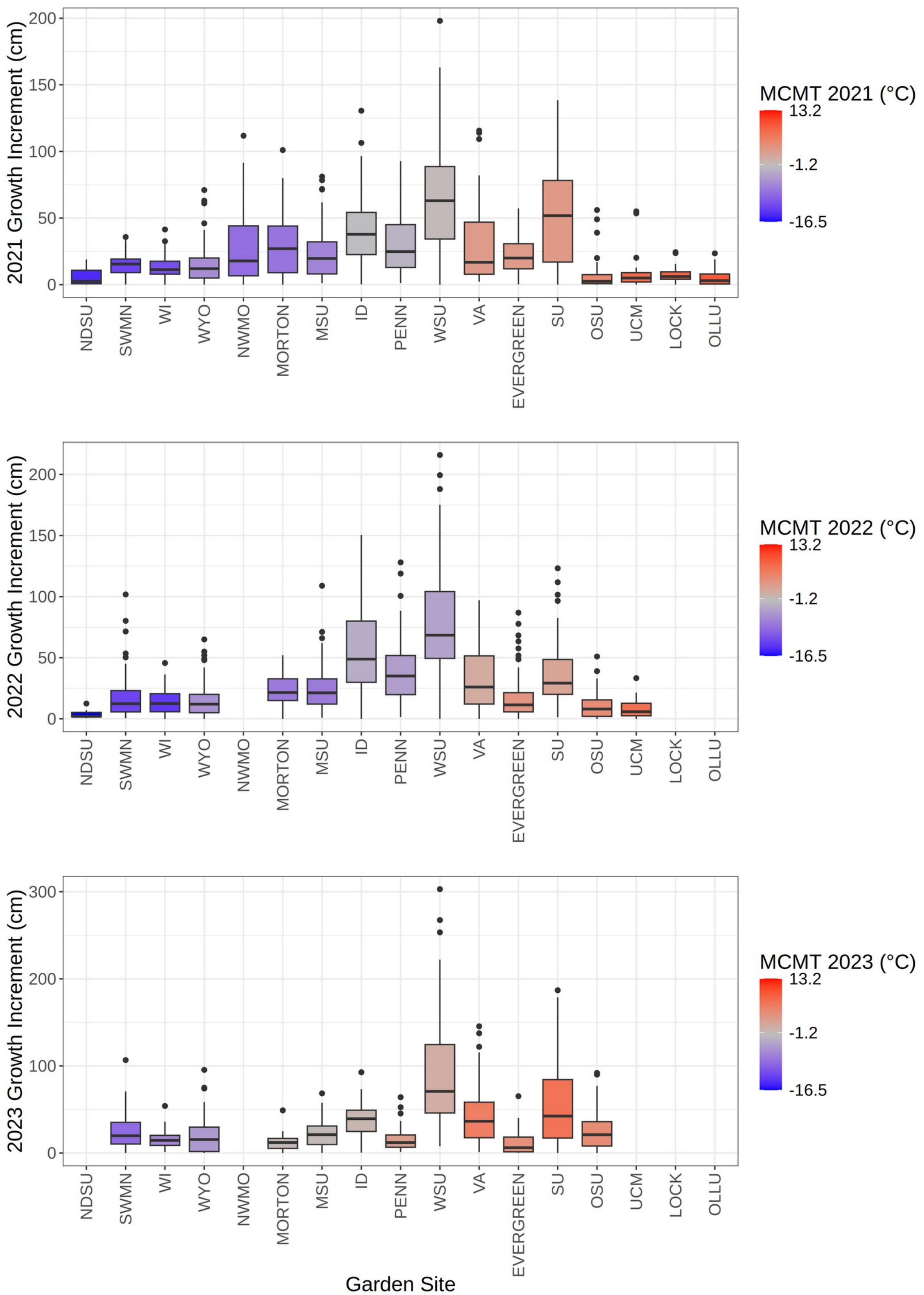

Figure S2. Variation in growth increment in centimeters (cm) across common gardens sites for each year of measurement. Gardens are ordered along the x-axis by their mean coldest month temperature (MCMT) averaged from 2020-2023. Garden boxplots are colored by the MCMT (°C) of that year.

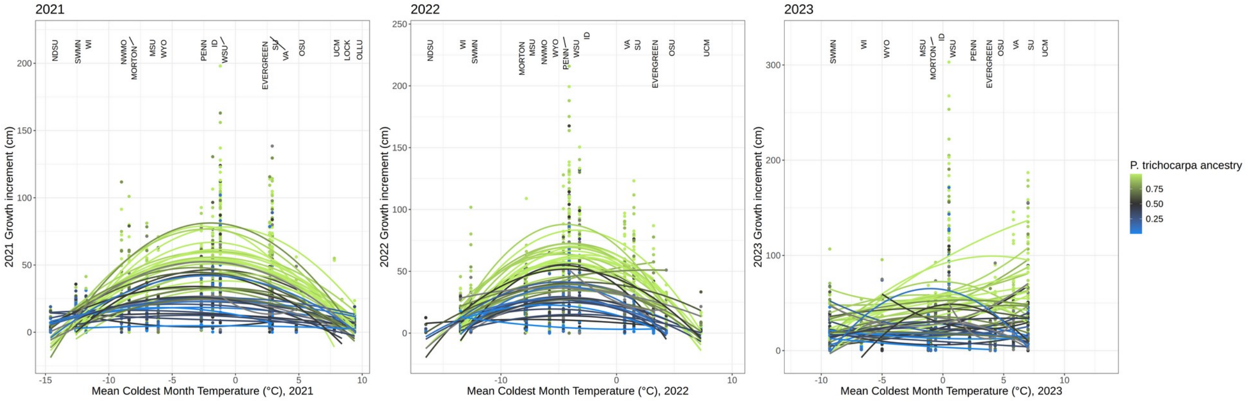

Figure S3. The genotype-specific response of yearly growth increment to garden mean coldest month temperature (MCMT) for three separate years (2021-2023), fit using a quadratic model of garden MCMT. Line colors indicate species ancestry, with green representing *Populus trichocarpa* ancestry and blue representing *P. balsamifera* ancestry. Text labels indicate the MCMT value of each garden for that year.

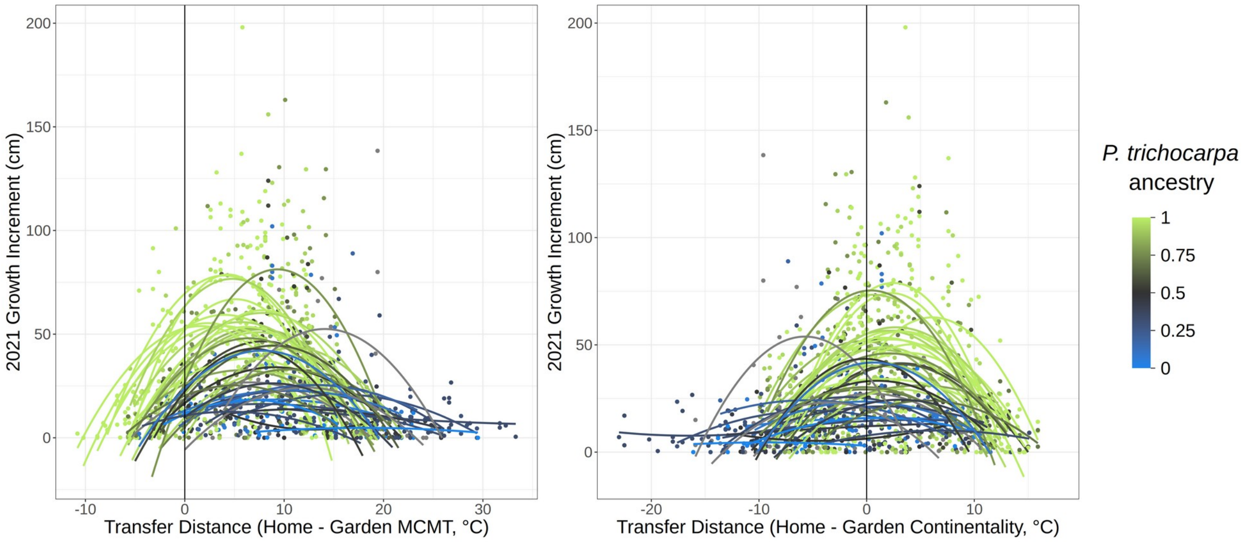

Figure S4. Response of 2021 growth increment to transfer distance, or the difference between home and common garden climate. Lines show the response of each genotype and are fitted using a quadratic model with the lm function in R. Points show raw data for each individual. Colors indicate species ancestry, with green representing *Populus trichocarpa* ancestry and blue representing *P. balsamifera* ancestry. Genotypes generally have higher growth in environments with warmer mean coldest month temperature (MCMT) and similar continentality (TD) to their climate of origin.

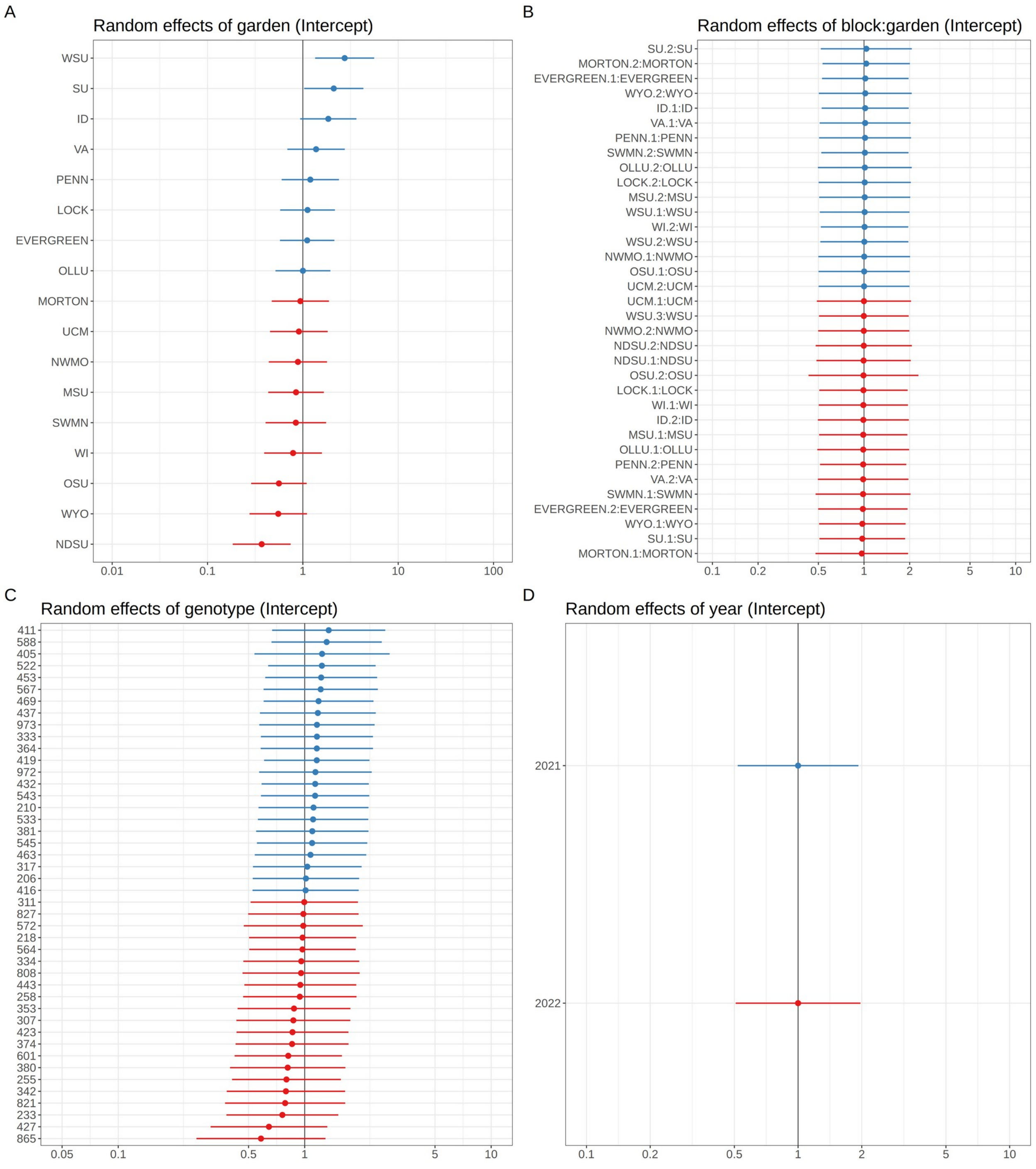

Figure S5. Estimates of random intercepts of Model 1, predicting growth increment, for A) garden site, B) block nested within garden, C) genotype, and D) year. Points depict deviation of estimates from the mean intercept, with estimates greater or less than one indicating a higher (blue) and lower (red) intercept, respectively. Lines show the 95% confidence intervals.

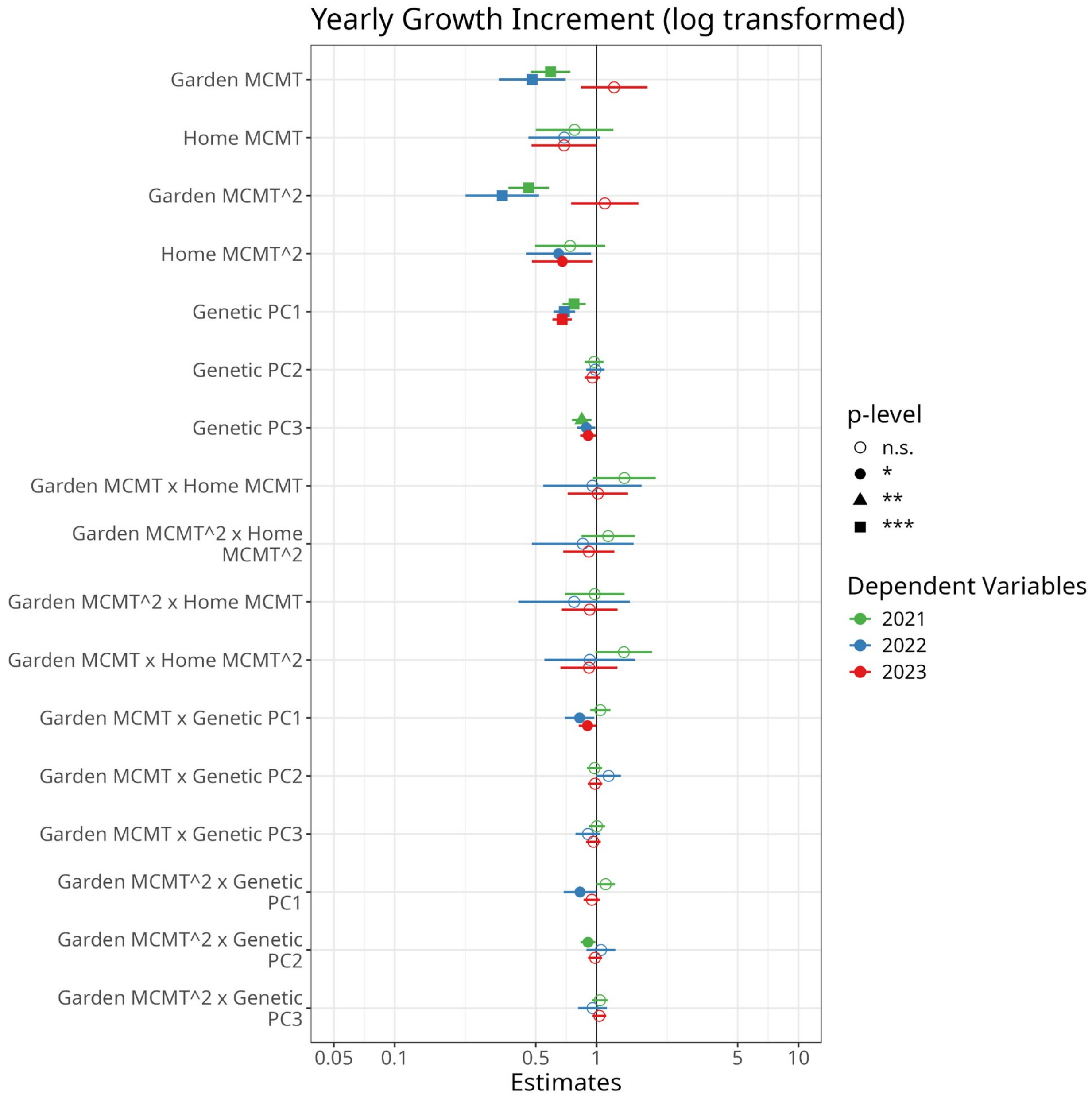

Figure S6. Forest plot of models for genetic and environmental factors explaining growth and mortality of admixed *Populus trichocarpa* and *P. balsamifera* genotypes planted in common gardens, with each year modeled separately. Effects include principal components (PCs) of genetic data, mean coldest month temperature (MCMT) of the garden and home site, and their interactions (as in Figure 2, main text). A model was fit to the subset of data from each year by modifying Model 1 to drop the random effects of year and individual, as there is one measurement per year per individual. Points indicate standardized effect estimates and lines represent 95% confidence intervals. Colors represent the model for each year, and point shapes

represent p-values calculated using a type II Wald chi square test test, and represented as follows: *<0.05, **<0.01, ***<0.001.

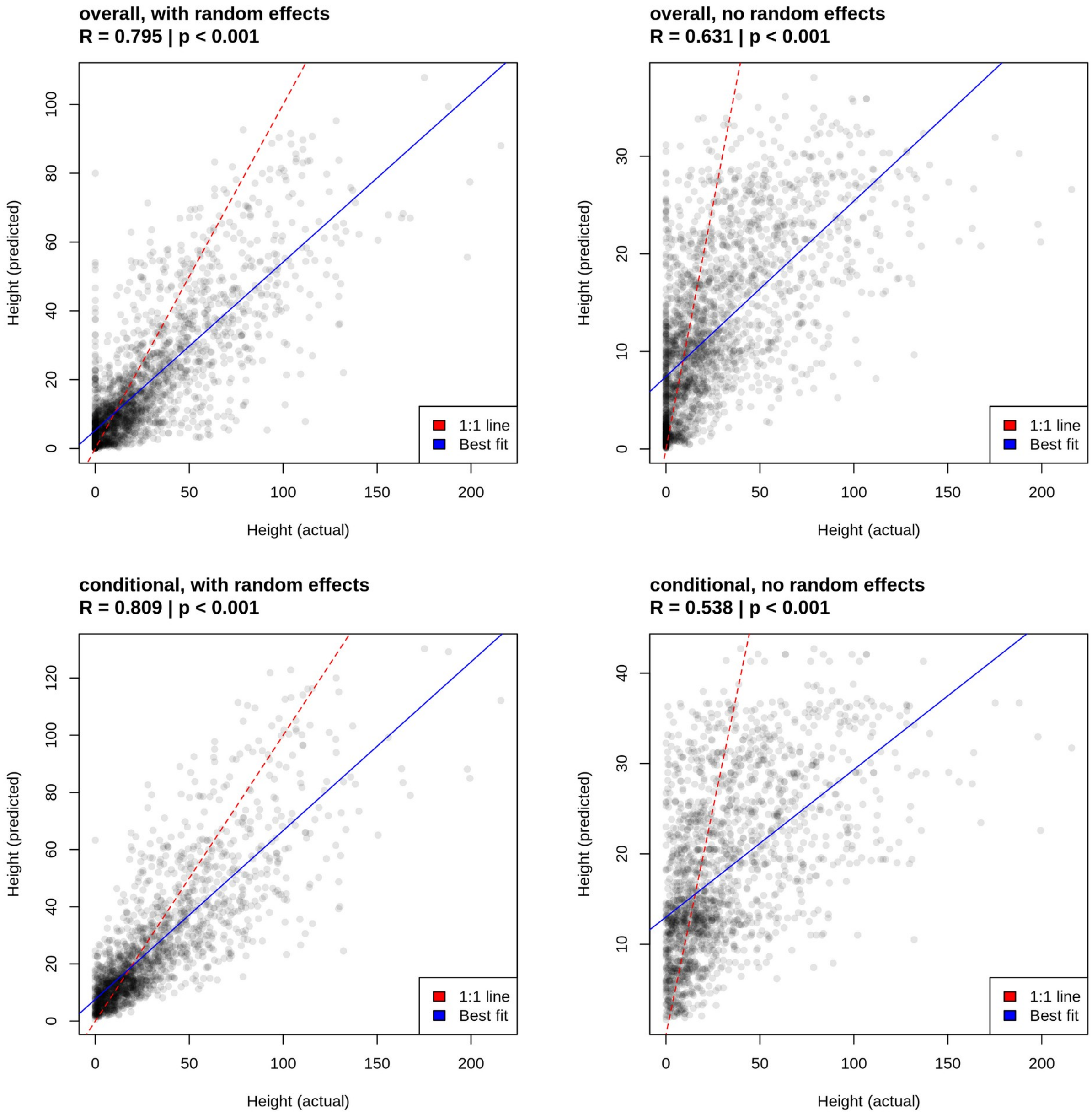

Figure S7. Correlation between actual and predicted growth for individuals across all 17 garden sites and two years, with the Pearson correlation (R) and its p-value (p), across four categories of predictions from the model. Results are shown for heights predicted from the overall model, including growth and mortality, with dead individuals having a height of zero (top) and for the conditional component representing growth in the surviving individuals (bottom). When the random effects of genotype, garden, and block are included (left) predictions are more closely correlated with actual heights compared to when they were excluded (right). Best fit lines have a lower slope than the 1:1 line, indicating that the model underpredicts height for taller individuals.

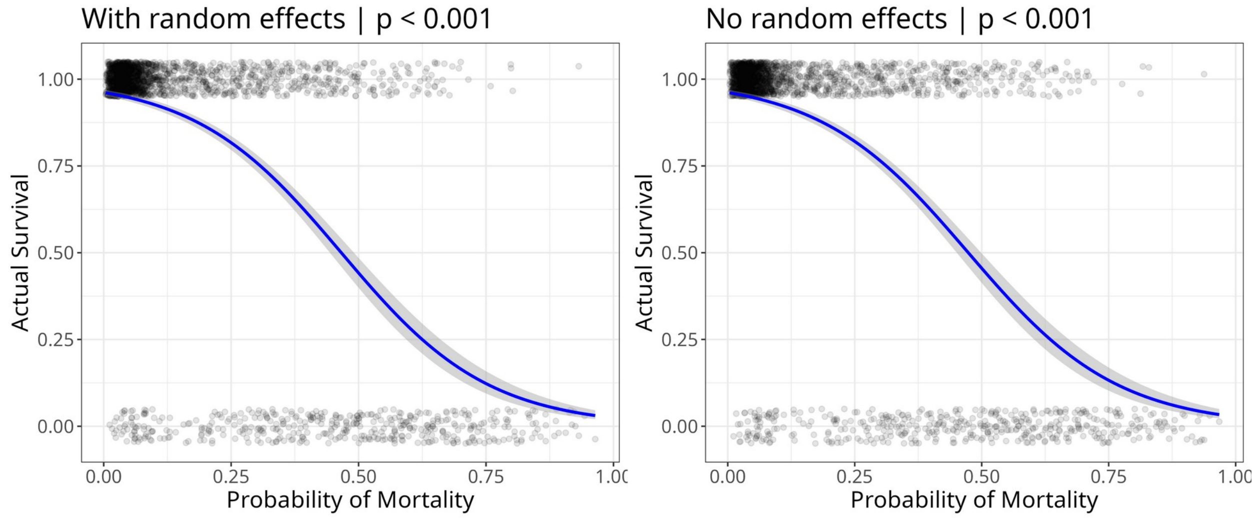

Figure S8. Relationship between actual survival and the probability of mortality predicted from Model 1, including all individuals across 17 garden sites and two years. The logistic relationship was modeled using a generalized linear model with a binomial link function, and the p-values for each model are shown. Survival was measured as a binary variable, with 1 indicating a living tree and 0 indicating a dead tree. Left plot shows the relationship when the random effects of genotype, garden, and block are included, and right when random effects are excluded.

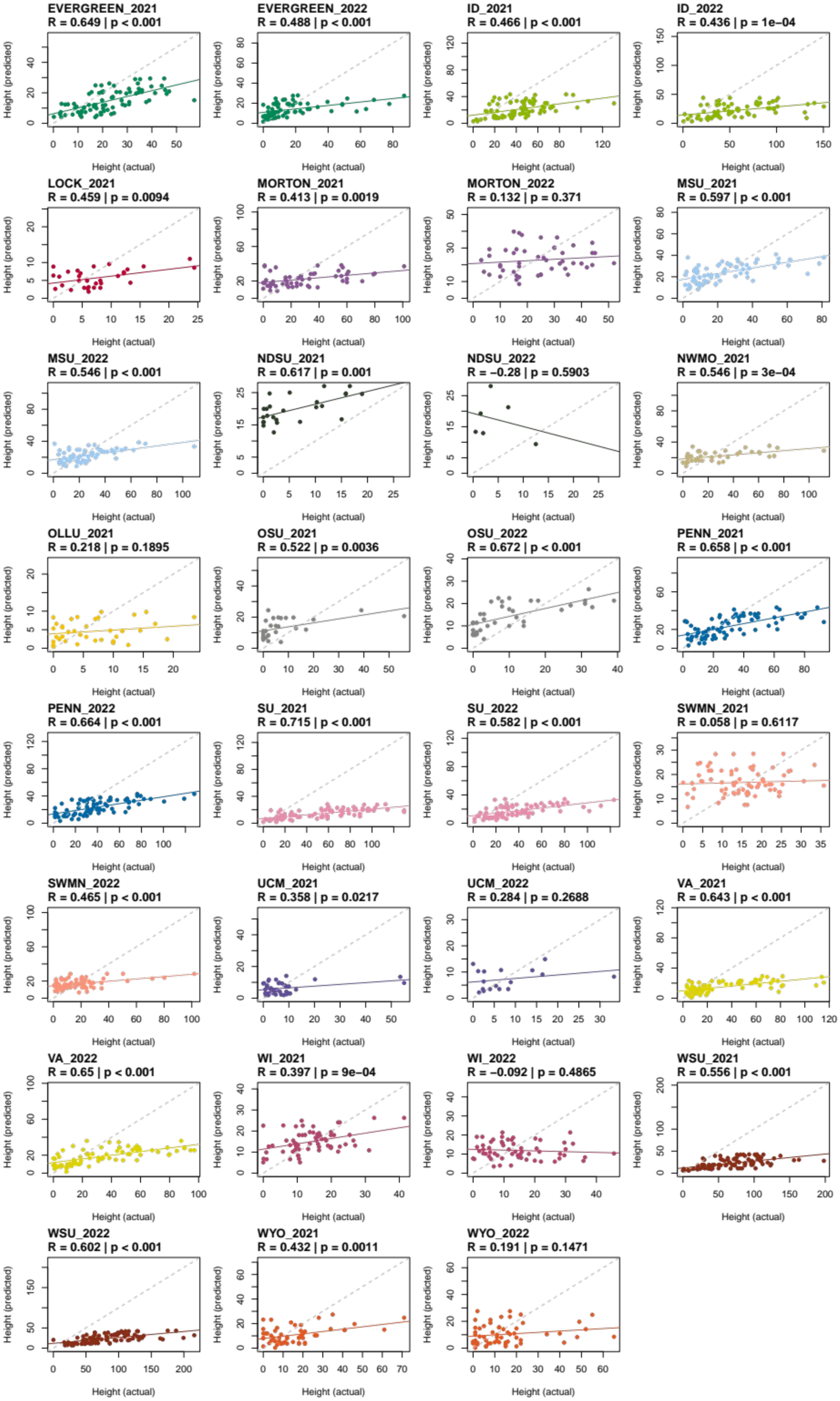
EVERGREEN_2021
R = 0.649 | p < 0.001
EVERGREEN_2022
R = 0.488 | p < 0.001
ID_2021
R = 0.466 | p < 0.001
ID_2022
R = 0.436 | p = 1e−04
LOCK_2021
R = 0.459 | p = 0.0094
MORTON_2021
R = 0.413 | p = 0.0019
MORTON_2022
R = 0.132 | p = 0.371
MSU_2021
R = 0.597 | p < 0.001
MSU_2022
R = 0.546 | p < 0.001
NDSU_2021
R = 0.617 | p = 0.001
NDSU_2022
R = −0.28 | p = 0.5903
NWMO_2021
R = 0.546 | p = 3e−04
OLLU_2021
R = 0.218 | p = 0.1895
OSU_2021
R = 0.522 | p = 0.0036
OSU_2022
R = 0.672 | p < 0.001
PENN_2021
R = 0.658 | p < 0.001
PENN_2022
R = 0.664 | p < 0.001
SU_2021
R = 0.715 | p < 0.001
SU_2022
R = 0.582 | p < 0.001
SWMN_2021
R = 0.058 | p = 0.6117
SWMN_2022
R = 0.465 | p < 0.001
UCM_2021
R = 0.358 | p = 0.0217
UCM_2022
R = 0.284 | p = 0.2688
VA_2021
R = 0.643 | p < 0.001
VA_2022
R = 0.65 | p < 0.001
WI_2021
R = 0.397 | p = 9e−04
WI_2022
R = −0.092 | p = 0.4865
WSU_2021
R = 0.556 | p < 0.001
WSU_2022
R = 0.602 | p < 0.001
WYO_2021
R = 0.432 | p = 0.0011
WYO_2022
R = 0.191 | p = 0.1471
Height (predicted)
Height (actual)

Figure S9. Correlation between actual and predicted growth in centimeters for each garden and year from leave-one-out cross validation predictions in gardens, in which predictions were made for the garden using a model trained on the other gardens. Pearson correlation (R) and p-values (p) for the correlation are shown. Solid lines indicate the best fit line; grey dotted lines indicate the one-to-one line.

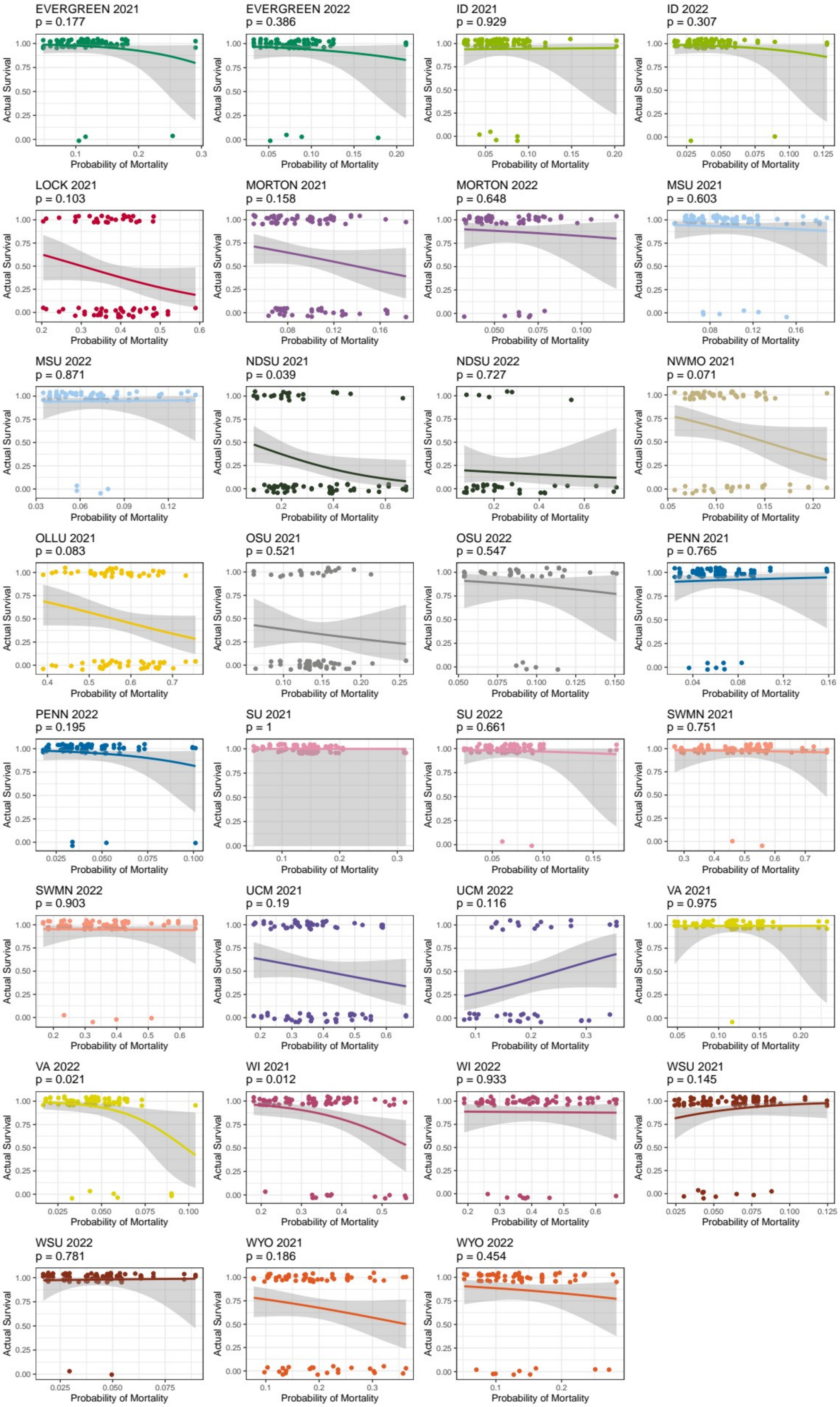
EVERGREEN 2021
p = 0.177
EVERGREEN 2022
p = 0.386
ID 2021
p = 0.929
ID 2022
p = 0.307
LOCK 2021
p = 0.103
MORTON 2021
p = 0.158
MORTON 2022
p = 0.648
MSU 2021
p = 0.603
MSU 2022
p = 0.871
NDSU 2021
p = 0.039
NDSU 2022
p = 0.727
NWMO 2021
p = 0.071
OLLU 2021
p = 0.083
OSU 2021
p = 0.521
OSU 2022
p = 0.547
PENN 2021
p = 0.765
PENN 2022
p = 0.195
SU 2021
p = 1
SU 2022
p = 0.661
SWMN 2021
p = 0.751
SWMN 2022
p = 0.903
UCM 2021
p = 0.19
UCM 2022
p = 0.116
VA 2021
p = 0.975
VA 2022
p = 0.021
WI 2021
p = 0.012
WI 2022
p = 0.933
WSU 2021
p = 0.145
WSU 2022
p = 0.781
WYO 2021
p = 0.186
WYO 2022
p = 0.454
Actual Survival
Probability of Mortality

Figure S10. Relationship between predicted probability of mortality and actual survival for each garden and year from leave-one-out cross validation predictions in gardens, in which predictions were made for the garden using a model trained on the other gardens. The logistic relationship was modeled using a generalized linear model with a binomial link function, and the p-values for each model are shown.

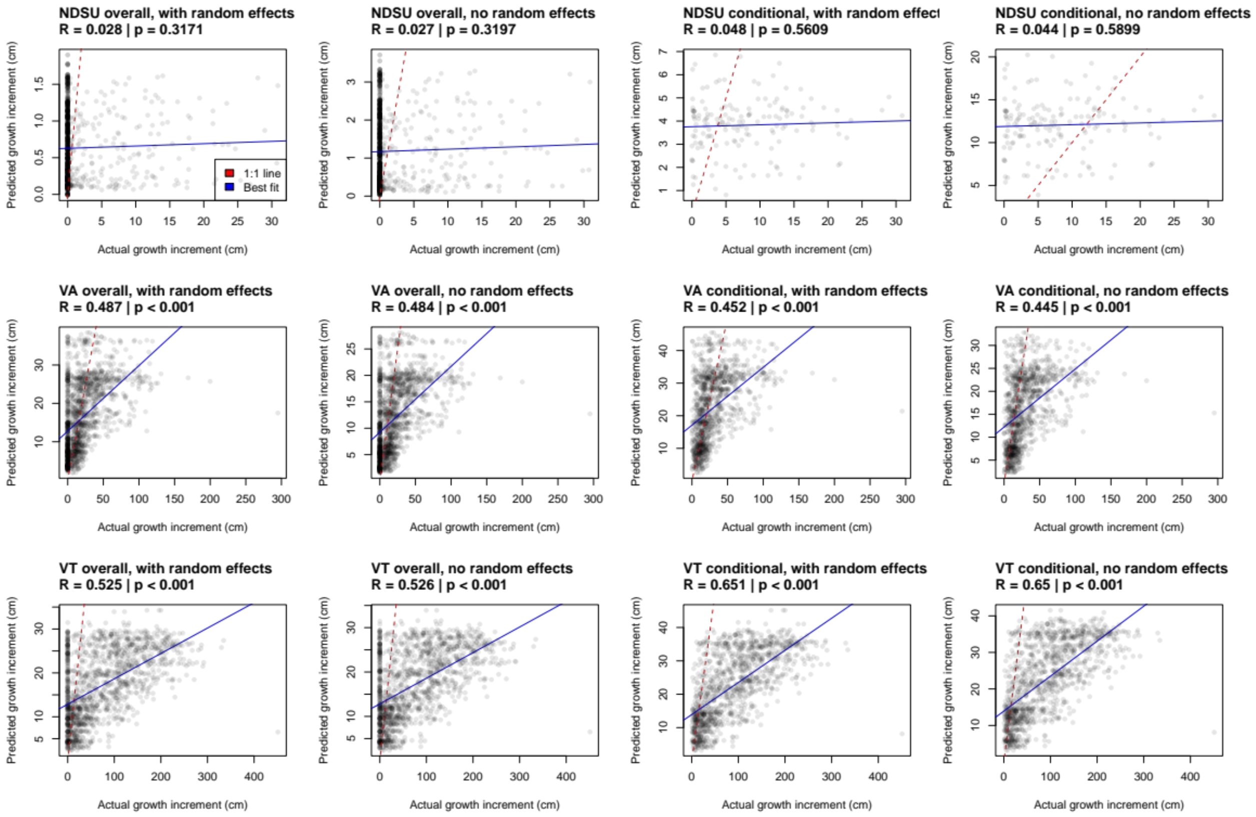

Figure S11. Correlation between actual and predicted growth for individuals for the three maxi garden sites, with the Pearson correlation (R) and its p-value (p), across four categories of predictions from the model. The overall model represents both growth and mortality, with dead individuals having a height of zero, and the conditional component represents growth in the surviving individuals. Results are also presented for predictions including or excluding the random effects of genotype, garden, and block.

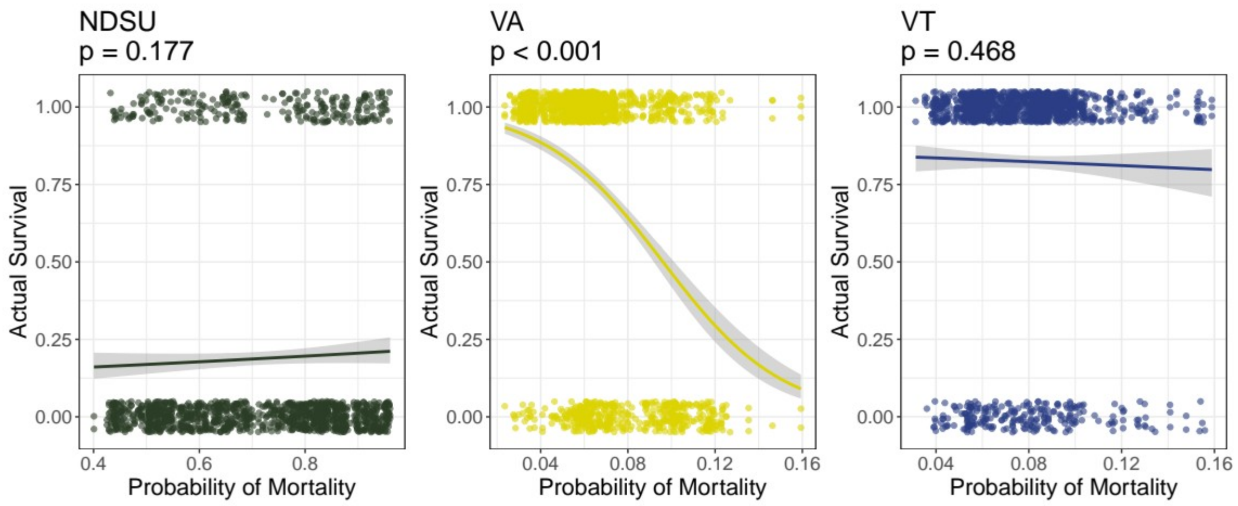

Figure S12. Relationship between predicted probability of mortality and actual survival for the three maxi garden sites, in which predictions were made for the garden using a model trained on the 17 mini gardens. The logistic relationship was modeled using a generalized linear model with a binomial link function, and the p-values for each model are shown.

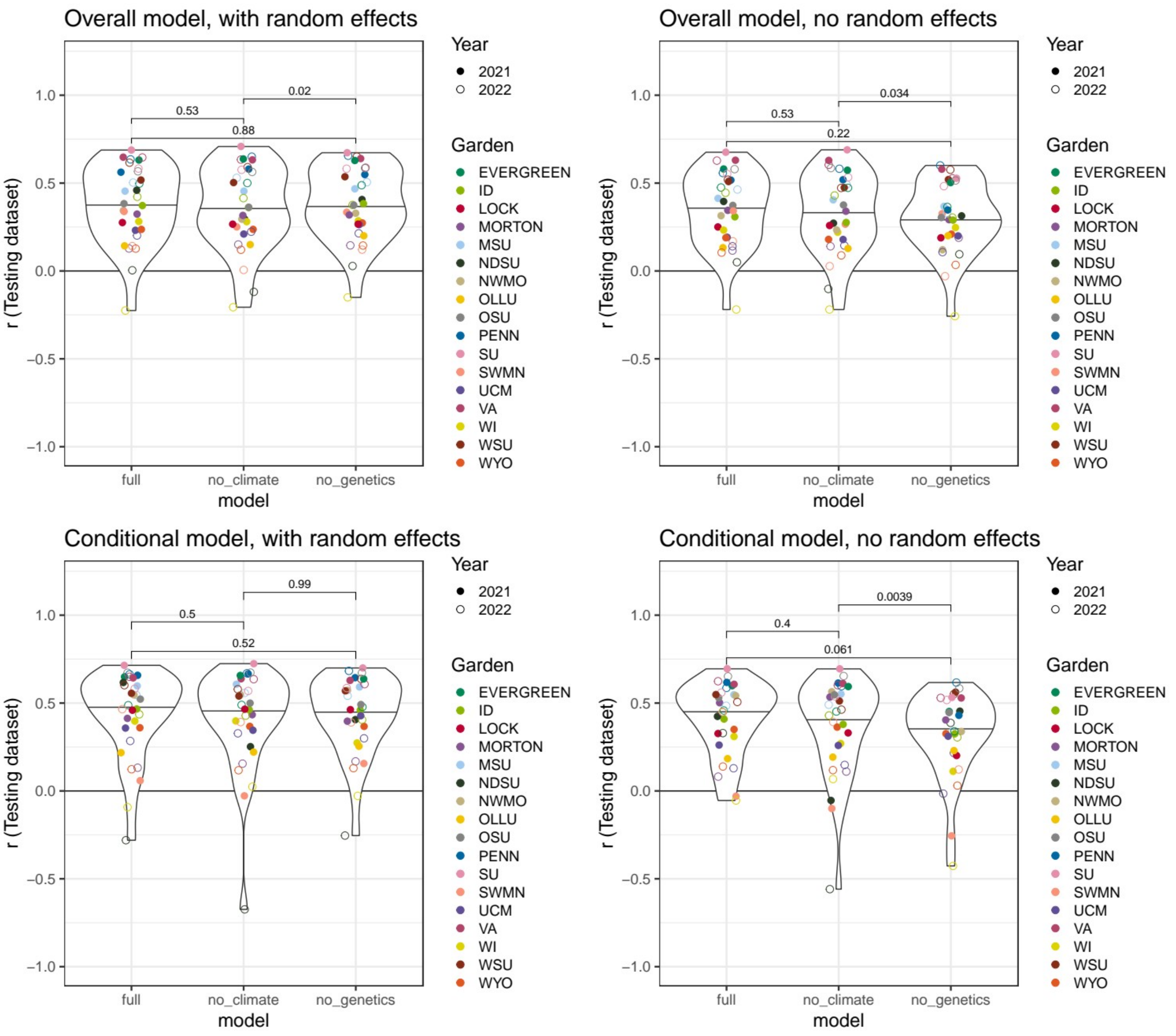

Figure S13. Comparison of performance among models predicting the yearly growth increment for admixed *Populus trichocarpa* and *P. balsamifera* genotypes planted in common garden environments across four categories of predictions from the model. Performance was compared among the full model and those excluding provenance climate as represented by mean coldest month temperature (MCMT), or genetic structure represented by genetic PCs (Table 1). Model predictive ability was estimated as the Pearson correlation (r) between predicted and observed growth increments for each garden and year predicted using the leave-one-out models, in which growth increments were predicted for a single year and garden using a model trained on other gardens. Predictions for the overall model represent both growth and mortality, and the conditional model represents growth in the surviving individuals. Predictions when the random effects of genotype, garden, and block are included or excluded. P-values are shown above brackets for each pairwise model comparison using a paired Wilcoxon test.

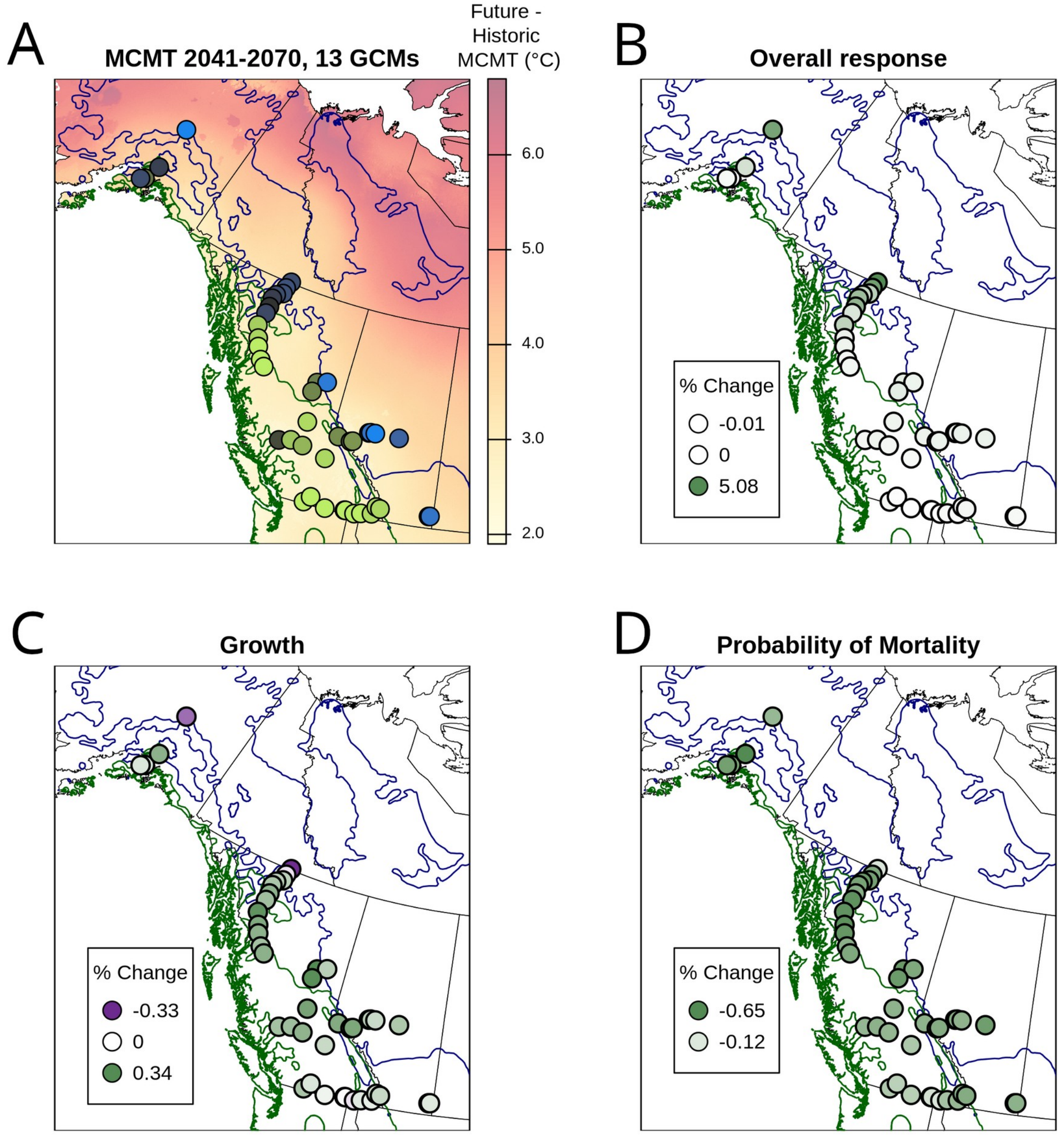

Figure S14. Predicted increases in mean coldest month temperature (MCMT) and the resulting predicted change in fitness metrics for each genotype at its home site, based on their norm of reaction (Figure 5). (A) Change in MCMT between the periods of 1961-1990 and 2041-2070 under SSP 2-45. Points show collected genotypes and their species ancestry, with green representing *Populus trichocarpa* ancestry and blue representing *P. balsamifera* ancestry. (B-D) Fitness changes for each genotype at their home site, indicated by points. Green indicates increased fitness, purple indicates decreased fitness, and white indicates no change; minimum and maximum values for each metric are shown in the legend. C) Predictions for the full model, combining growth and mortality. C) Predictions for changes in yearly growth increment predicted from the conditional model component. D) Predictions for changes in the probability of mortality predicted from the zero-inflated model component. All predictions are based on changes in MCMT between the periods of 1961-1990 and 2041-2070 under shared socioeconomic pathway (SSP) 2-45.

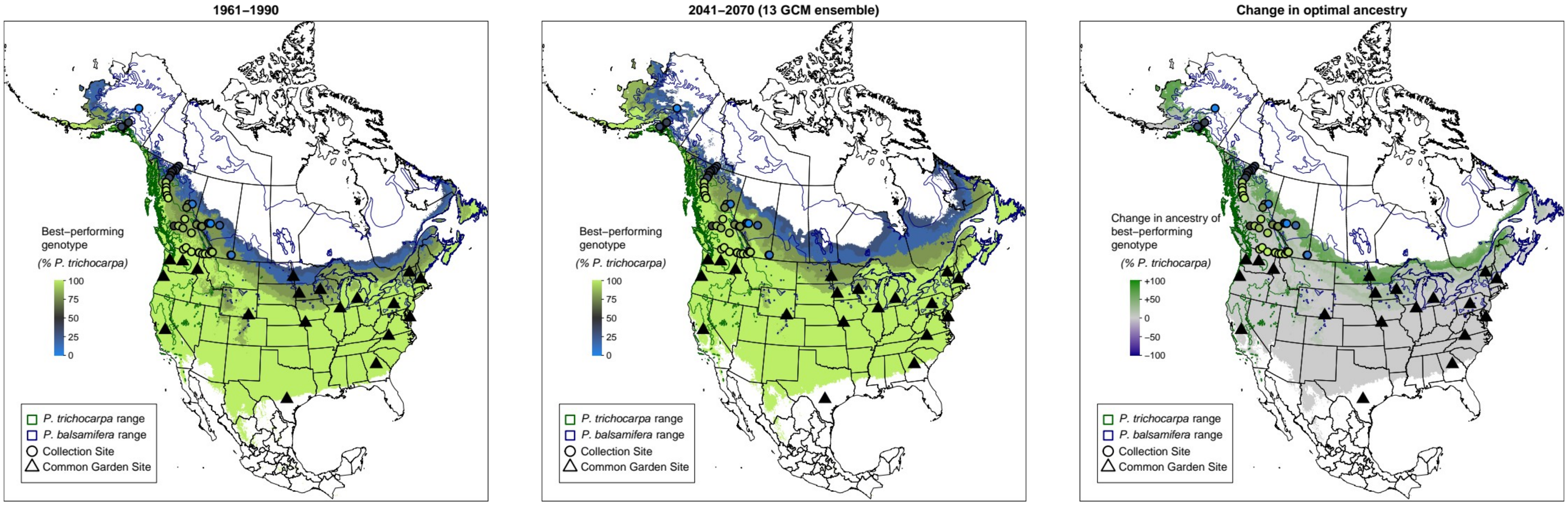

Figure S15. Maps showing the species ancestry of the studied genotype which is predicted to have highest fitness (as measured by growth and mortality) in that location under historic and future climate, indicated by the color of the base layer, across the ranges of both species and the common garden sites included in this study. "Change in optimal ancestry" indicates the change in optimal species ancestry between future and historic climates, indicating regions where increased *P. trichocarpa* ancestry is expected to be beneficial. See Figure 6 (main text) for corresponding figures inset to the sampled hybrid zone.

Figure S16 (attached as a separate file). Predicted reaction norms across values of mean coldest month temperature (MCMT) as in Figure 5a, with each genotype plotted separately (solid curve) with its MCMT of origin (vertical dotted line), allowing performance to be compared among the "local" genotype (MCMT of origin is the same as MCMT at planting site) and all other genotypes. Under the "local vs foreign" criterion of local adaptation, the genotype originating from a particular environment should outperform other genotypes in that environment (Kawecki & Ebert, 2004).

Supplementary Tables

Table S1. List of common garden sites, abbreviations, and years measured. Sites that have a maxi garden or both a maxi and mini garden are indicated by parentheses ("maxi" and "both", respectively). All other sites are mini gardens.

Common Garden Site	Abbreviation	City	State	Years Measured
Evergreen State	EVERGREEN	Olympia	WA	2021-2023
Lockerly Arboretum	LOCK	Milledgeville	GA	2021
Michigan State University	MSU	East Lansing	MI	2021-2023
Missouri Arboretum	NWMO	Maryville	MO	2021-2022
Morton Arboretum	MORTON	Lisle	IL	2021-2023
North Dakota State University (both)	NDSU	Fargo	ND	2021-2022
Oregon State University	OSU	Corvallis	OR	2021-2023
Our Lady of the Lake University	OLLU	San Antonio	TX	2021
Pennsylvania State University	PENN	State College	PA	2021-2023
Salisbury University Arboretum	SU	Salisbury	MD	2021-2023
Southwest Minnesota State University	SWMN	Marshall	MN	2021-2023
UC Merced	UCM	Merced	CA	2021-2022
University of Idaho	ID	Moscow	ID	2021-2023
University of Wisconsin - Eau Claire	WI	Eau Claire	WI	2021-2023
University of Wyoming	WYO	Laramie	WY	2021-2023
University of Vermont (maxi)	VT	Burlington	VT	2021
Virginia Tech (both)	VA	Critz	VA	2021-2023
Washington State - Wenatchee	WSU	Wenatchee	WA	2021-2023

Table S2. Differences between Akaike information criterion (AIC) scores when different climate variables are used. MCMT has the lowest AIC score. The ‘dAIC’ column shows the increase in AIC score from the best model, and ‘df’ indicates degrees of freedom. Produced by the AICtab function from R package bbmle (version 1.0.25.1).

	**dAIC**	**df**
MCMT	0	41
DD_0	3.3	41
DD_18	6.1	41
EMT	12.3	41
MAT	23.6	41
NFFD	37.2	41
Eref	48.5	41
TD	54.9	41
eFFP	81.1	41
DD5	88.6	41
MAP	88.7	41
SHM	91.9	41
FFP	93.1	41
CMD	97.6	41
EXT	99.4	41
RH	99.4	41
bFFP	102	41
DD18	105.2	41
AHM	105.7	41
MSP	107	41
PAS	109.6	41
MWMT	114	41

Table S3. Effects of the linear mixed-effect model predicting yearly growth increment: home and garden MCMT, the square terms of MCMT, genetic PCs 1-3, and their interactions. Effects are shown for the conditional component testing each factor's effect on growth, and for the zero-inflated component testing the effect on the probability of mortality. Standardized beta coefficients are calculated by dividing the estimate for each predictor by its standard deviation to enable the relative effect sizes of predictors to be compared. Model table was produced by the tab_model function in the sjPlot (version 2.8.17) package in R.

**Conditional Model**						
**Predictors**	**Estimates**	**std. Beta**	**CI**	**standardized CI**	**p**	**std. p**
(Intercept)	2.85	2.73	2.13 – 3.57	2.48 – 2.98	**<0.001**	**<0.001**
Garden MCMT	-0.28	-0.53	-0.83 – 0.28	-0.74 – -0.31	0.33	**<0.001**
Home MCMT	-0.42	-0.27	-1.57 – 0.72	-0.65 – 0.11	0.468	0.169
Garden MCMT^2	-0.83	-0.46	-1.53 – -0.13	-0.74 – -0.18	**0.02**	**0.001**
Home MCMT^2	-0.54	-0.33	-1.14 – 0.06	-0.68 – 0.01	0.077	0.06
Genetic PC1	-0.33	-0.3	-0.46 – -0.21	-0.41 – -0.18	**<0.001**	**<0.001**
Genetic PC2	0.05	-0.02	-0.06 – 0.16	-0.12 – 0.07	0.35	0.66
Genetic PC3	-0.18	-0.15	-0.29 – -0.08	-0.24 – -0.05	**0.001**	**0.003**
Garden MCMT × Home MCMT	0.64	0.21	-0.27 – 1.55	-0.09 – 0.51	0.168	0.168
Garden MCMT^2 × Home MCMT^2	0.23	0.11	-0.33 – 0.79	-0.16 – 0.37	0.427	0.427
Home MCMT × Garden MCMT^2	-0.1	-0.03	-1.19 – 0.98	-0.32 – 0.27	0.855	0.855
Garden MCMT × Home MCMT^2	0.38	0.21	-0.10 – 0.85	-0.06 – 0.49	0.121	0.121
Garden MCMT × Genetic PC1	-0.01	-0.01	-0.12 – 0.10	-0.11 – 0.09	0.86	0.86
Garden MCMT × Genetic PC2	0.01	0.01	-0.07 – 0.09	-0.06 – 0.08	0.796	0.796
Garden MCMT × Genetic PC3	-0.03	-0.03	-0.11 – 0.05	-0.10 – 0.05	0.487	0.487
Garden MCMT^2 × Genetic PC1	0.04	0.03	-0.08 – 0.16	-0.06 – 0.12	0.521	0.521
Garden MCMT^2 × Genetic PC2	-0.1	-0.07	-0.20 – 0.00	-0.15 – 0.00	0.052	0.052
Garden MCMT^2 × Genetic PC3	0.04	0.03	-0.07 – 0.14	-0.05 – 0.10	0.487	0.487
(Intercept)	0.69	0.69	0.65 – 0.73	0.65 – 0.73		
**Zero-Inflated Model**						
**Predictors**	**Estimates**	**std. Beta**	**CI**	**standardized CI**	**p**	**std. p**
(Intercept)	-2.99	-2.03	-5.18 – -0.80	-2.81 – -1.26	**0.008**	**<0.001**

Garden MCMT	0.05	0.74	-1.31 – 1.42	0.10 – 1.37	0.94	**0.023**
Home MCMT	-0.07	0.24	-3.61 – 3.47	-0.74 – 1.21	0.97	0.636
Garden MCMT^2	2.08	1.34	0.11 – 4.05	0.72 – 1.95	**0.039**	**<0.001**
Home MCMT^2	0.15	0.2	-1.68 – 1.97	-0.69 – 1.08	0.876	0.663
Genetic PC1	0.14	0.08	-0.24 – 0.51	-0.21 – 0.36	0.475	0.604
Genetic PC2	-0.21	-0.17	-0.54 – 0.12	-0.42 – 0.07	0.217	0.163
Genetic PC3	-0.11	0.03	-0.44 – 0.22	-0.22 – 0.28	0.506	0.803
Garden MCMT × Home MCMT	-1.54	-0.51	-3.69 – 0.62	-1.22 – 0.21	0.164	0.164
Garden MCMT^2 × Home MCMT^2	-0.27	-0.13	-1.89 – 1.35	-0.89 – 0.64	0.741	0.741
Home MCMT × Garden MCMT^2	0.15	0.04	-3.02 – 3.33	-0.82 – 0.91	0.925	0.925
Garden MCMT × Home MCMT^2	-0.88	-0.5	-2.04 – 0.28	-1.16 – 0.16	0.136	0.136
Garden MCMT × Genetic PC1	0.04	0.03	-0.20 – 0.28	-0.18 – 0.25	0.756	0.756
Garden MCMT × Genetic PC2	0.04	0.03	-0.16 – 0.24	-0.14 – 0.21	0.71	0.71
Garden MCMT × Genetic PC3	-0.02	-0.02	-0.22 – 0.18	-0.20 – 0.16	0.843	0.843
Garden MCMT^2 × Genetic PC1	-0.06	-0.05	-0.39 – 0.27	-0.30 – 0.20	0.711	0.711
Garden MCMT^2 × Genetic PC2	0.07	0.05	-0.24 – 0.37	-0.17 – 0.27	0.671	0.671
Garden MCMT^2 × Genetic PC3	0.2	0.15	-0.10 – 0.50	-0.08 – 0.38	0.192	0.192

Table S4. Effect sizes of the random effects (τ) of genotype, garden, block nested within garden, year, and individual and their sample sizes (N) for Model 1. Table was produced by the tab_model function in the sjPlot package (version 2.8.17) in R.

**Random Effects**	
σ2	0.69
τ00 genotype	0.05
τ00 block:Garden	0
τ00 Garden	0.24
τ00 year	0
τ00 indiv	0.06
N genotype	44
N block	35
N Garden	17
N year	2
N indiv	1448
Observations	2308
Marginal R2 / Conditional R2	0.348 / NA
